# Recent progress to construct calixarene-based polymers using covalent bonds: synthesis and applications

**DOI:** 10.1039/d0ra05707j

**Published:** 2020-09-03

**Authors:** Reza Zadmard, Fahimeh Hokmabadi, Mohammad Reza Jalali, Ali Akbarzadeh

**Affiliations:** Chemistry and Chemical Engineering Research Center of Iran Iran zadmard@ccerci.ac.ir

## Abstract

The combination of supramolecular chemistry and polymer sciences creates a great possibility to afford calixarene-based polymers offering unique features and applications. The enhancement of calixarene's versatility in this manner has made chemists better able to achieve different objectives in host–guest chemistry. The calixarene-based polymers can be divided into covalent polymers and supramolecular polymers regarding the interactions. Although there are several studies available on the calixarene-based supramolecular polymers, there is a paucity of studies on the calixarene-based covalent polymers. In this paper, the most recent developments and applications of the calixarene-based covalent polymers in the last two decades have been reviewed. We have particularly focused on the polymers, including those where the calixarene molecules have been used as macromonomers and polymerize through covalent bonds. Moreover, covalent polymers or solid supports functionalized with calixarenes are highlighted as well.

## Abstract 2

The combination of supramolecular chemistry and polymer sciences creates a great possibility to afford calixarene-based polymers offering unique features and applications.

## Graphical abstract

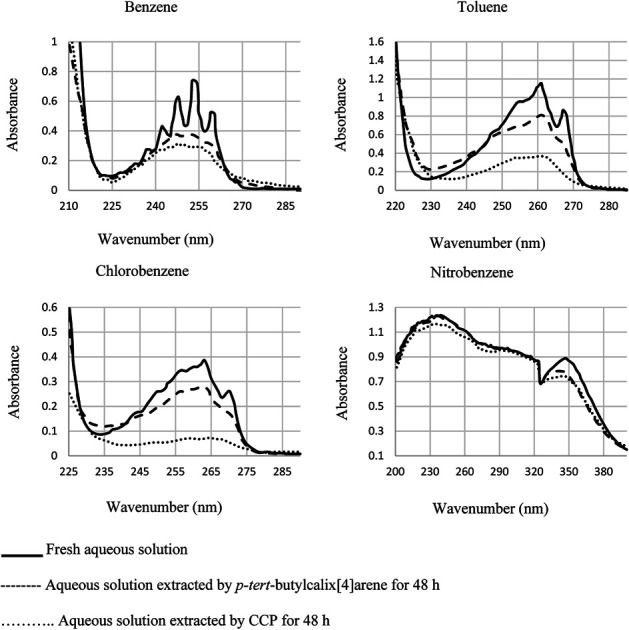

## 1. Introduction

Calixarene is a typical motif in supramolecular chemistry. Following crown ethers and cyclodextrins, calixarenes are third-generation host molecules. It is easy to synthesize calixarenes using condensation reactions of phenols with aldehydes in a desirable yield. Actually, calixarene was first discovered in 1872 by Adolph von Bayer and then in the late 1970s, Gutsche *et al.* improved the chemistry of phenol-formaldehyde products and coined the term “Calixarene”.^1,2^ As a result of special geometry and the availability of active groups in calixarenes, it is possible to use these compounds for various purposes.^3^ The calixarene structure includes a polar rim, a non-polar rim, and a hydrophobic cavity. The rims can be selectively functionalized to provide analyte-selectivity or to facilitate polymerization. The internal cavity of calixarene can host different guest molecules; therefore, calixarene-based polymers are suitable for manipulating selectivity in separation sciences. Because of these structural characteristics, calixarenes have been incorporated into polymers, but mostly as side-chain moieties. Several studies have been performed on the synthesis of calixarene-based covalent polymers. However, usually, calixarene-based polymers have been developed through either polymerization of calixarene-containing monomers or fixing of calixarene units on a polymeric matrix.^4–6^ The calixarene-based covalent polymers create an opportunity to produce materials with fantastic features. The polymers containing calixarene moieties on the main or side chains have the capacity to be used as chemical sensors,^7^ biosensors,^8^ selective membranes, catalysts,^9^ microelectronic devices,^10^ star-shaped polymers^11,12^ and drug delivery systems.^13^ Calixarene-centered amphiphilic copolymers synthesis and their assemblies in water had also investigated in recent studies.^14,15^ In this review, we describe the strategies for the synthesis of covalent polymers using calix[*n*]arene skeletons and their application aspects in two main parts, including “covalent polymers constructed by calixarene-containing monomers” and “functionalized polymers *via* calixarene moieties”. We begin by considering the recent studies in the synthesis of covalent polymers with calixarene-containing monomers which, have been categorized based on their applications, including iodine capture, removal of azo dyes, metal ions and toxic pollutants, molecular recognition, gels and hydrogels and other studies related to this kind of polymers. We then describe functionalized polymers *via* calixarene moieties such as Merrifield resin, Wang resin, silica gel, TentaGel, chitosan and other polymeric compounds. However, because of calixarenes unique three-dimensional structures and their ability to form non-covalent interactions, the previously reported review articles were mostly published based on applications and properties of the calixarene-based systems in the field of host–guest chemistry^16^ such as separation science,^17^ coordination polymers,^18^ organic–inorganic hybrid materials,^19^ calixarene derivatives as biosensors,^20^ supramolecular drug delivery systems,^21^*etc.*

## 2. Covalent polymers constructed by calixarene-containing monomers

It is supposed that covalent linkages of calixarenes in a polymer main chain demonstrate several interesting and applicable properties.^22^ In particular, the preparation of calixarene-containing polymers has two general synthetic methods. One is based on polymerization reactions of a functionalized calixarene monomers, and the other relied on the immobilization of the calixarene moiety on a polymeric matrix. In this part, we explain the most recent examples of polymerization of functionalized calixarene monomers plus the properties and applications of polymers achieved in this manner.

### 2.1 Iodine capture by calixarene-based covalent polymers

**Scheme 1:**
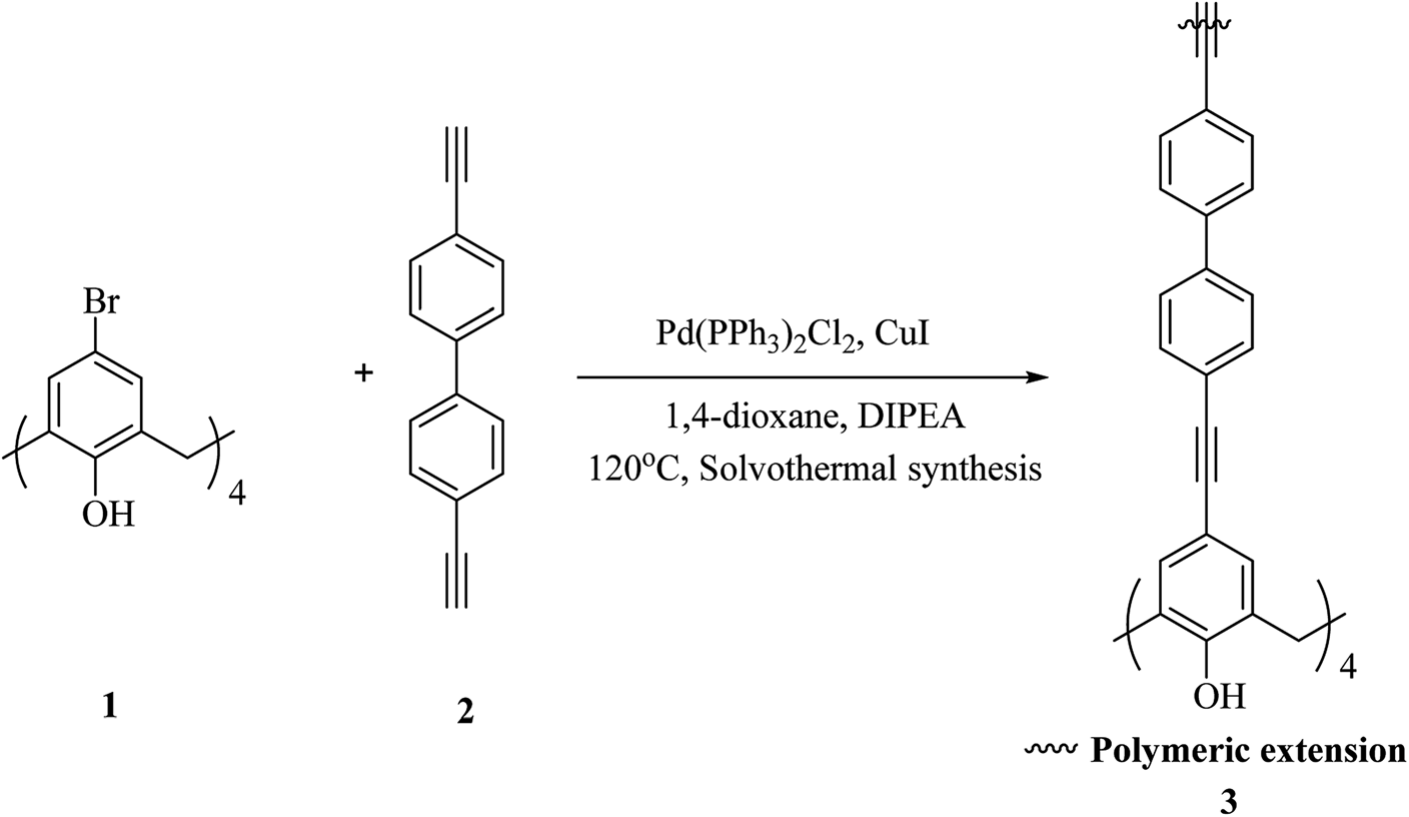
Calix[4]arene nanosheet 3 synthesis by Sonogashira–Hagihara cross-coupling reaction.

One of the key micronutrients is iodine, which has a key role in thyroid hormones producing. The high concentration of iodine is an environmental pollution which has critical ecological and health effect. Iodine is also an interesting radionuclide that creates serious risks to the public. So, it is important to sense and remove iodine from water and vapor phase and to this end, several calixarene-based polymers have been developed.^23,24^ These studies have introduced different types of adsorbents and molecules which have been examined to eliminate excess iodine from water and environment. For example, Shetty *et al.* introduced calix[4]arene-based 2D polymer 3 through Sonogashira–Hagihara cross-coupling reaction between tetra-bromo calix[4]arene tetrol 1 and 4,4′-diethynyl-1,1′-biphenyl 2 in anhydrous 1,4-dioxane (Scheme 1). The results of the characterization indicated that 2D polymer 3 was porous, covalent, and isolated as few-layer thick (3.52 nm) nanosheet. The polymer was examined as an I_2_ vapor adsorbent, and the findings were supported with a maximum capacity of 114 wt%. Moreover, it was easy to regenerate the material through washing with mild ethanol, and therefore it can be recycled with a very small loss of the efficiency.^25^

**Scheme 2:**
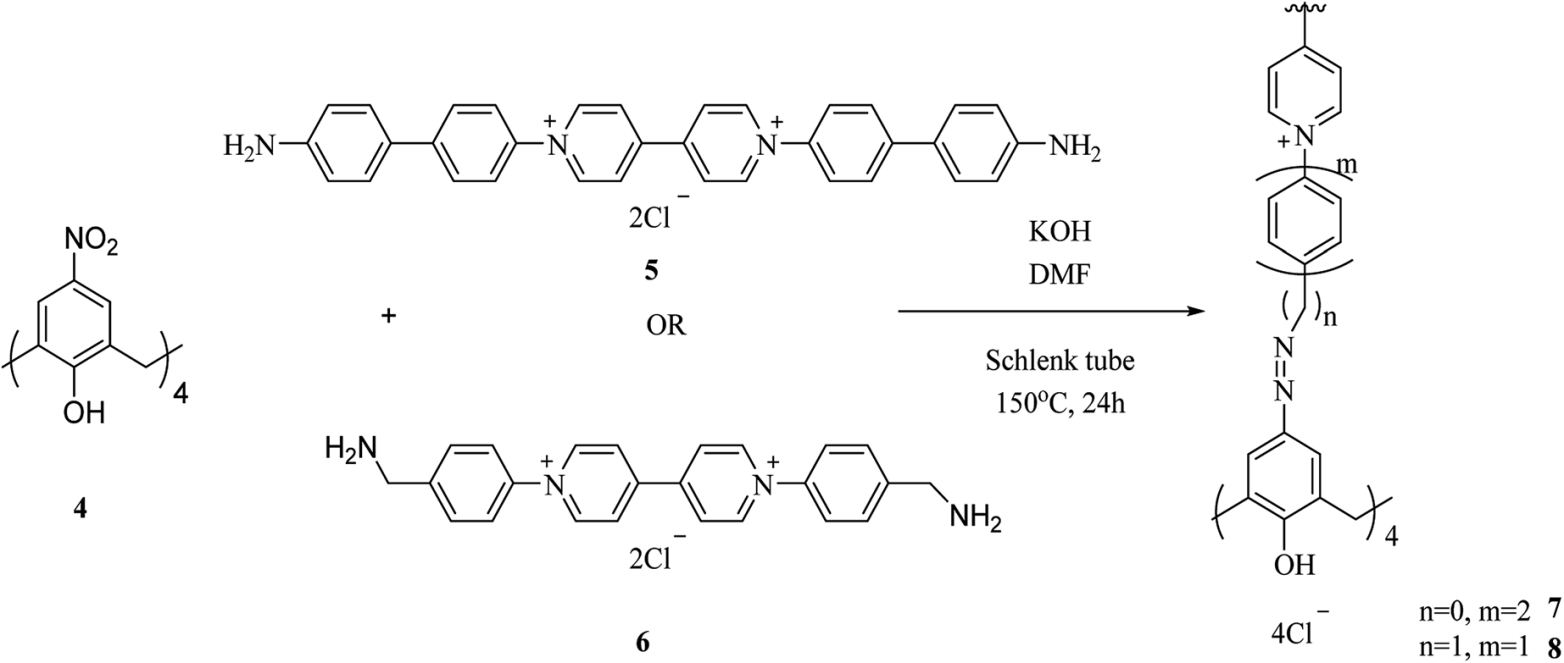
Preparation of copolymers 7 and 8.

**Scheme 3:**
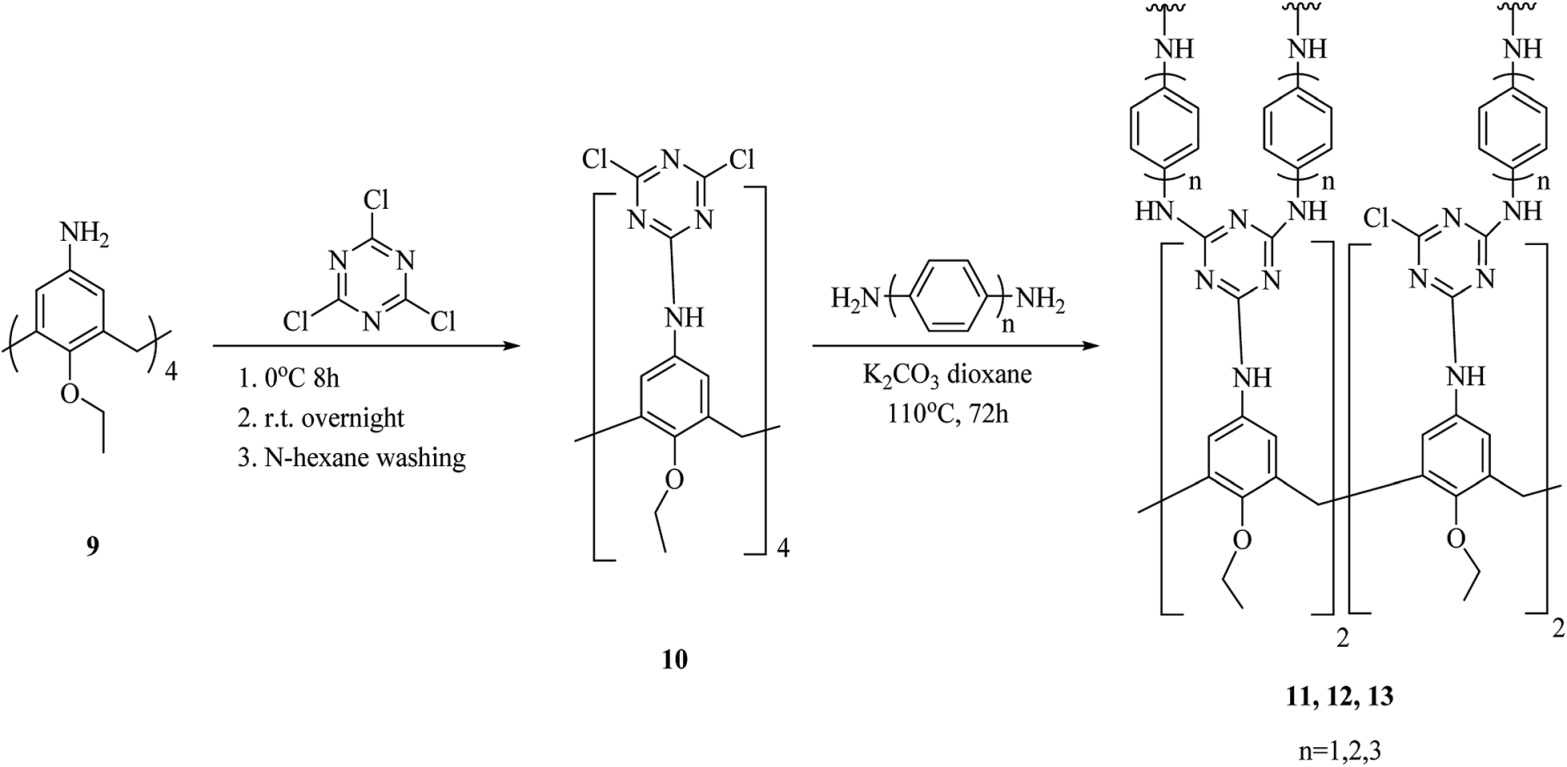
General synthesis of poly calix[4]arenes 11, 12, 13.

**Scheme 4:**
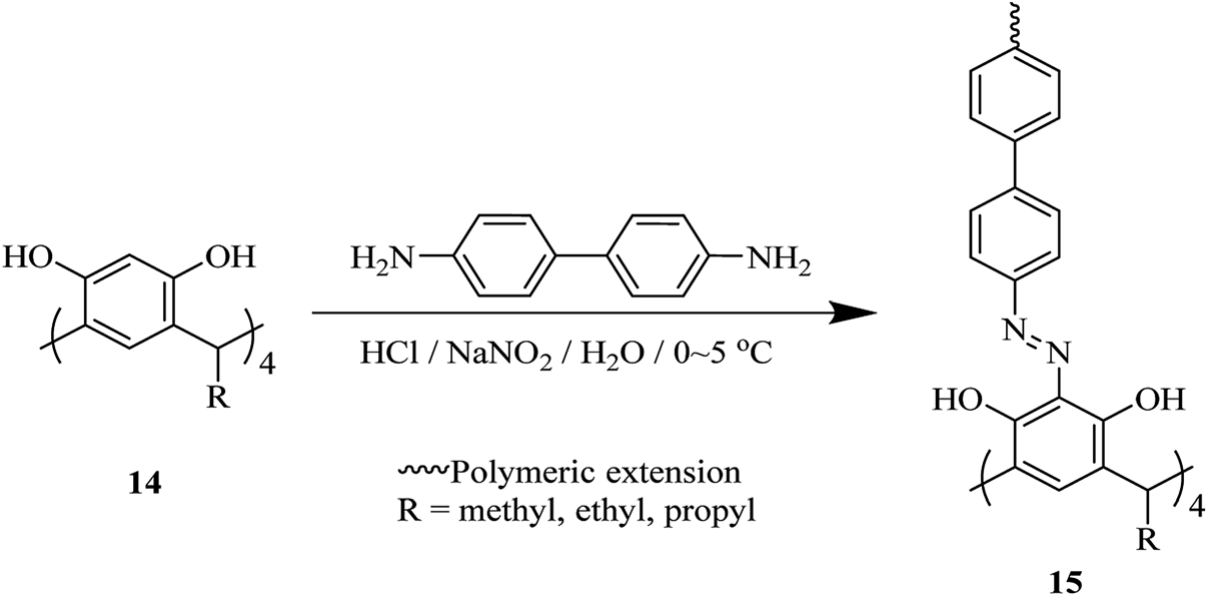
Typical design of diazocoupling reaction of 4,4′-biphenyldiamine and C-alkylcalix[4]resorcinarenes 14.

As another study covalent organic polymers 7 and 8 were also examined by Skorjanc *et al.* in terms of pollutant sponges. These polymers were produced using viologens 4, 5 and nitrocalix[4]arene 4 for iodine and toxic dyes capture. Scheme 2 illustrates the synthesis of the copolymers 7 and 8. Actually, they showed a capability of iodine sorption about 200% of their weight in iodine vapor and also could act as an effective sorbent for Congo red in aqueous solutions (pH range 2–10).^26^ An *et al.* introduced another case of iodine capture by using poly calix[4]arenes 11, 12 and, 13. The synthetic route to obtain these poly calix[4]arenes is shown in Scheme 3.^27^ Moreover, Su *et al.* studied azo-bridged calix[4]resorcinarene-based porous polymer 15 to capture volatile iodine (Scheme 4).^28^

**Scheme 5:**
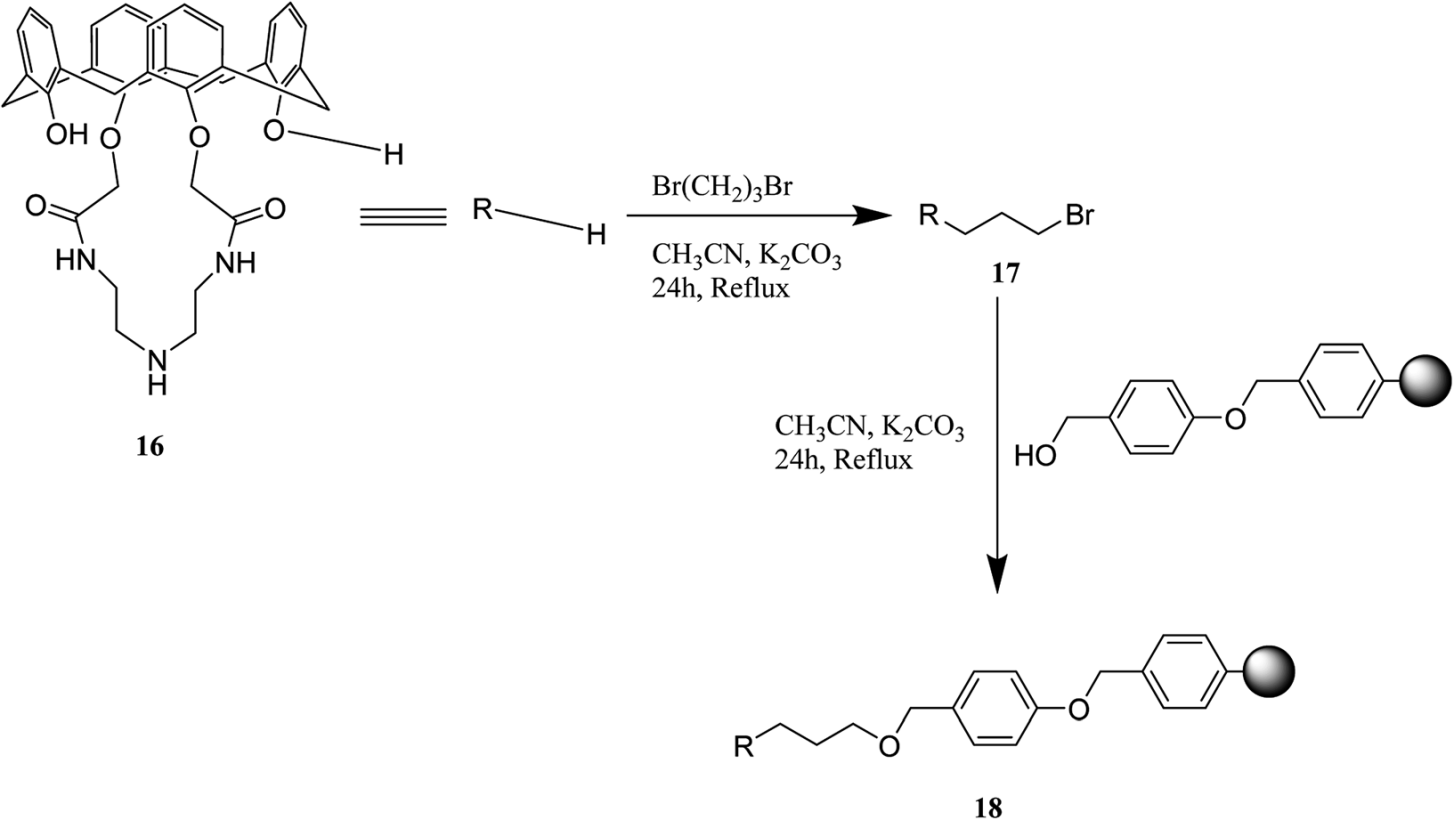
Preparation of polymer appended calix[4]amidocrown-5 18.

Sharma *et al.* also studied macroscopic identification of iodide using polymer attached calix[4]amidocrown resin. The polymer appended calix[4]amidocrown-5 18 is featured with special binding affinity for iodide at ppm-level (Scheme 5).^29^

### 2.2 Removal of azo dyes, metal ions and toxic pollutants

**Scheme 6:**
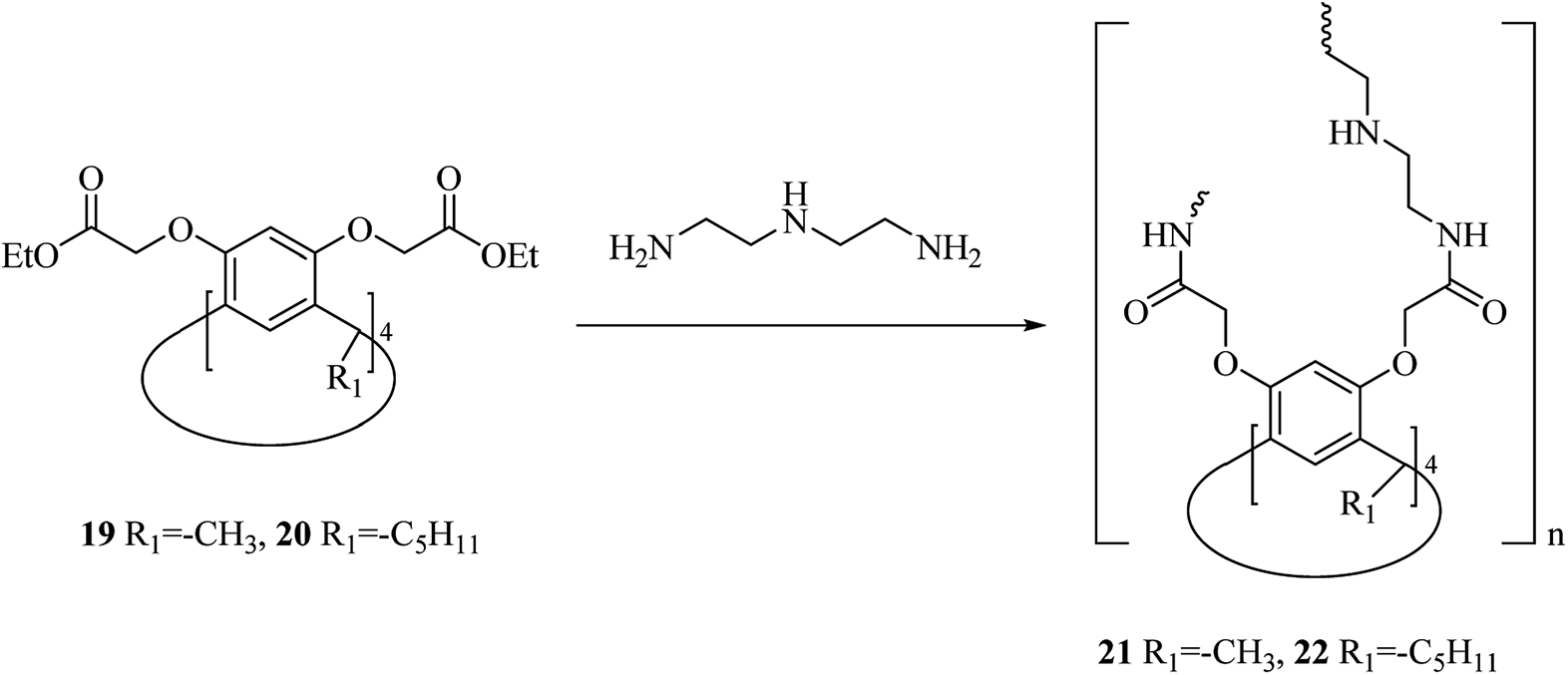
Synthesis rout of amidoamine calix[4]resorcinarene-based polymers 21, 22.

One of the main environmental problems imposed by industrial activities is removing toxic water-soluble pollutants and mainly non-biodegradable ones from wastewaters. Many biological and physicochemical methods are available for eliminating pollutants from wastewaters and among them, sorption is the most efficient method.^30,31^ A renowned class of synthetic receptor molecules is calixarenes. They are featured with intramolecular bow-shaped cavities that accommodate neutral and ionic molecular guests and metal ions by establishing selective and specific interactions of diverse origin.^32–34^ Shalaeva *et al.* introduced the synthesis of two novel polymers with amidoamine calix[4]resorcinarene units as macrocyclic sorbents towards three azo dyes including, methyl orange, acid orange and Congo red, all of which soluble in water. Both polymers 21 and 22 were produced using polycondensation of calix[4]resorcinarenes esters 19, 20 and diethylene triamine (Scheme 6).^35^

**Scheme 7:**
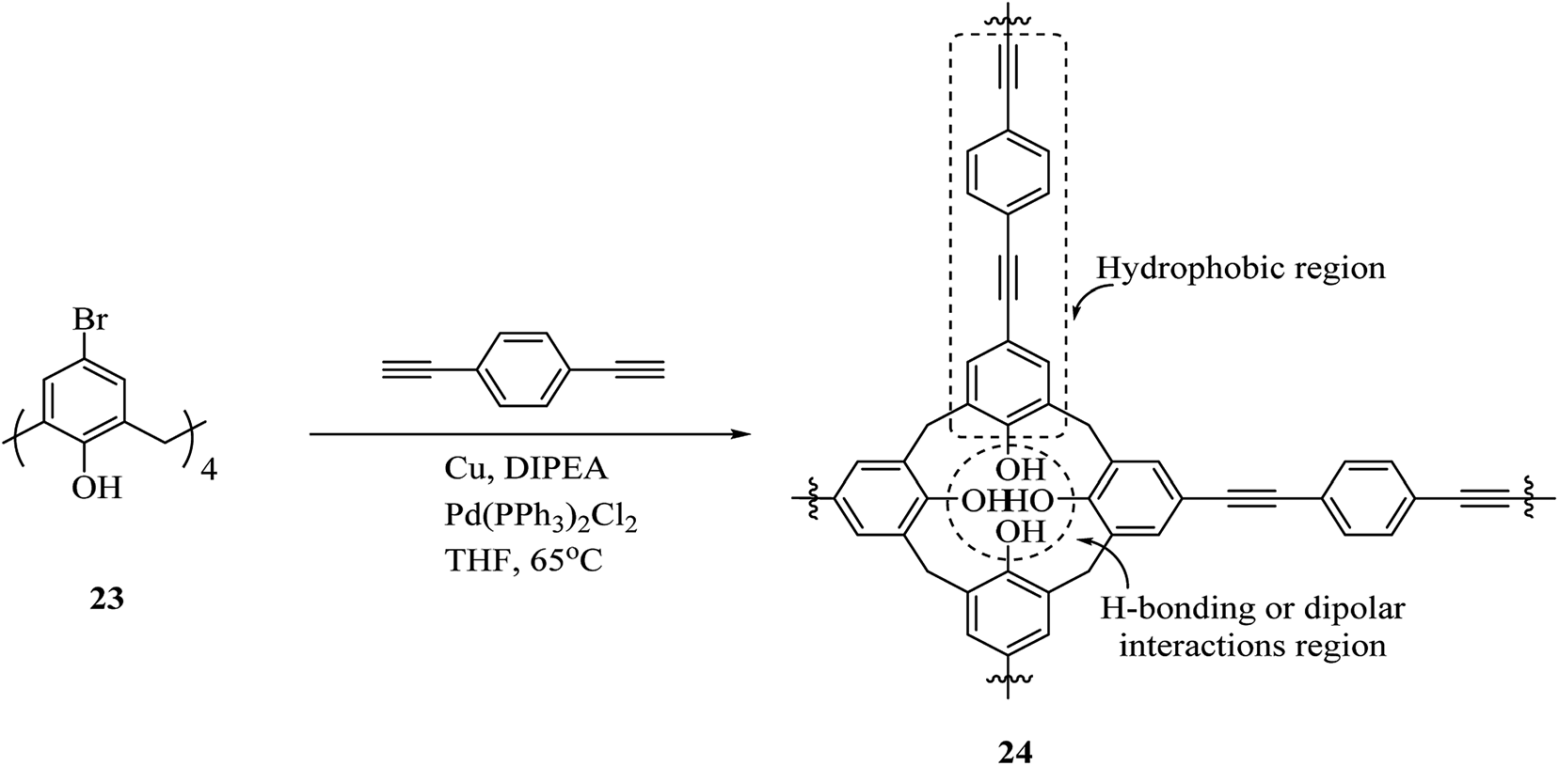
Porous calix[4]arene polymer 24 synthesis by Sonogashira–Hagihara reaction.

**Scheme 8:**
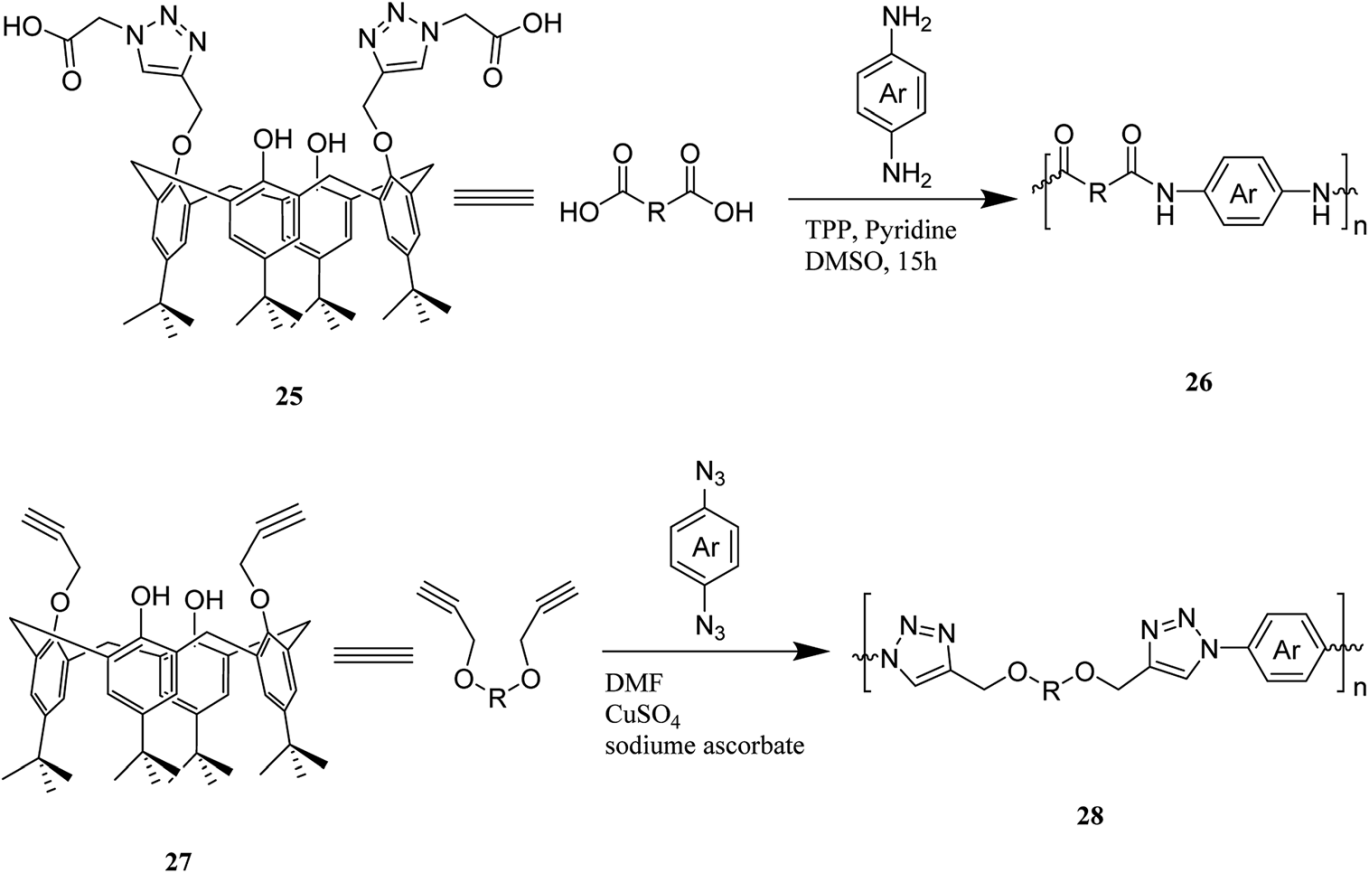
Synthetic pathway for the preparation of polycalix[4]amide 26 and polycalix[4]arene 28.

Additionally, an innovative calix[4]arene-based polymer 24 was effectively synthesized by Shetty *et al.* through palladium catalyzed Sonogashira–Hagihara cross-coupling of 1,4-diethynylbenzene and tetra-bromocalix[4]arene 23 (Scheme 7). The adsorption capacity in this polymer is significantly higher compared with other adsorbents such as activated carbon and, this may also remove the organic contaminant from water containing solvents, oils, and dyes. It is easy to produce the polymer and frequently use for sorption/desorption cycles with a notable performance.^36^ Lakouraj *et al.* introduced another way to remove the heavy metal ions from wastewater through solid–liquid extraction by polycalix[4]amide 26 and polycalix[4]arene 28 containing mesogenic triazole units (Scheme 8).^37^

**Scheme 9:**
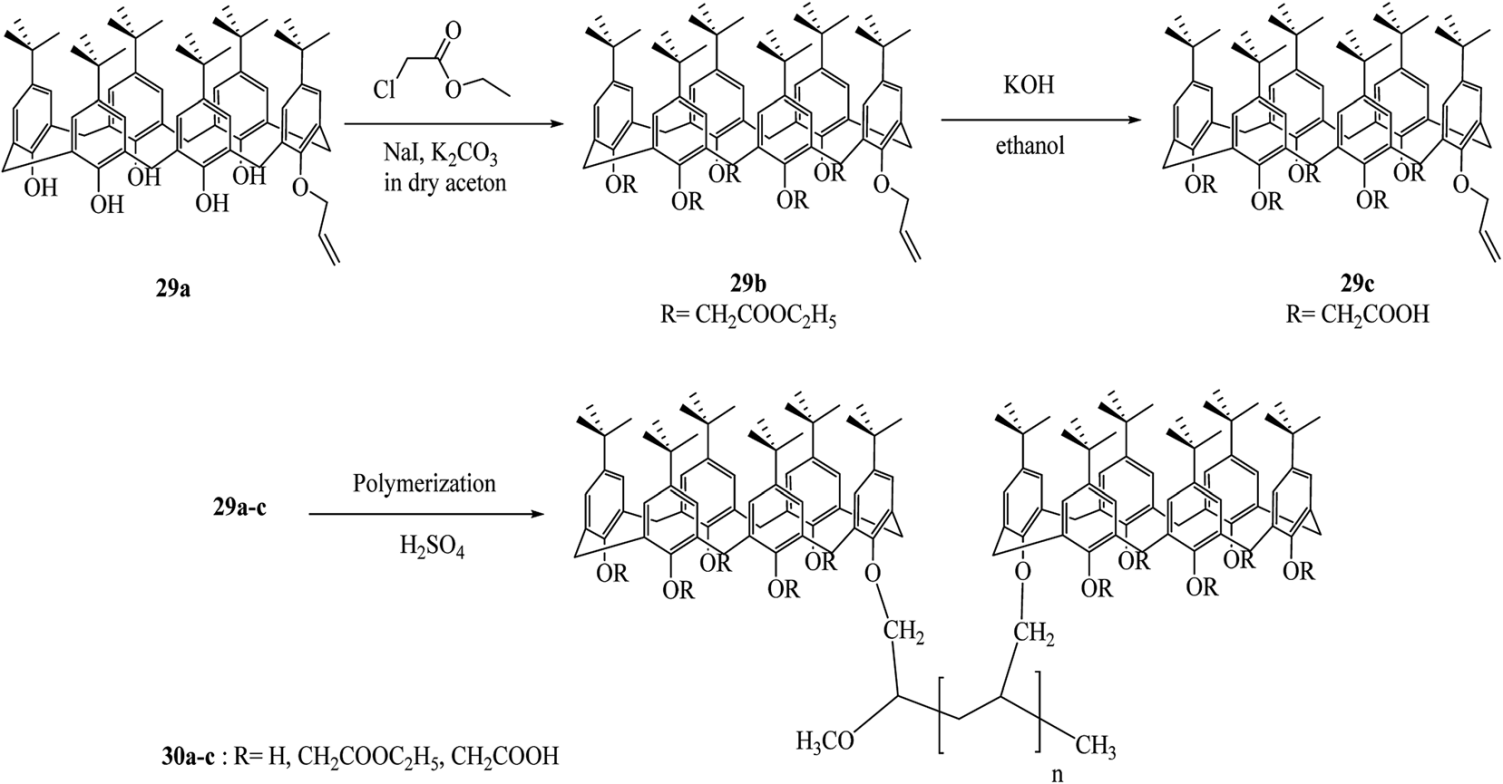
The schematic presentation of poly-calix[6]arenes 30a–c synthesis.

In a recent work, Prabawati *et al.* examined trapping of heavy metal cations by poly-monoallyloxy calix[6]arene 30a, poly-monoallyloxy penta-ester calix[6]arene 30b and poly-monoallyloxy penta-acid calix[6]arene 30c (Scheme 9). These three polymers easily trap the heavy metal cations such as Cd(ii), Cu(ii), and Cr(iii). It can be assumed that the availability of hydroxyl group (–OH) and channel-like structure of the polymers can be used as adsorbents for heavy metals.^38,39^

**Scheme 10:**
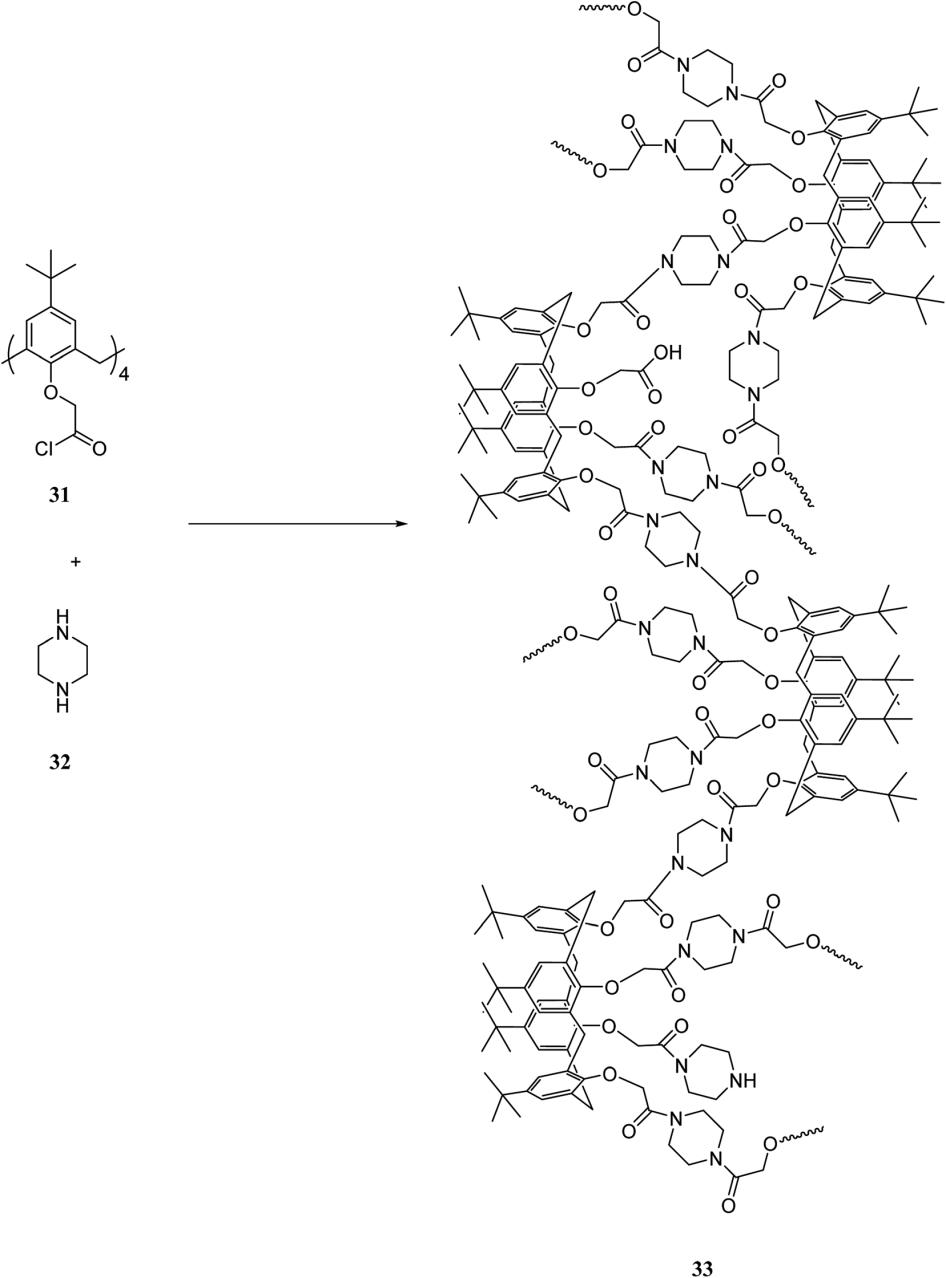
Structure and rout of synthesis of microcapsule 33.

Zhu *et al.* produced a calixarene-based microcapsule 33 using a non-agitation solution polymerization. This microcapsule showed the remarkable removing ability for metal cations (Scheme 10). Preparation of microcapsule was tested and proved by Fourier transform infrared spectrometry (FTIR) and the X-ray photoelectron spectroscopy (XPS). Furthermore, transmission electron microscope (TEM) and dynamic light scattering (DLS) illustrated that non-agitation solution polymerization is a better method to prepare a dispersion of microcapsule. This dispersion can extract heavy metal cations from their aqueous solution.^10^

**Scheme 11:**
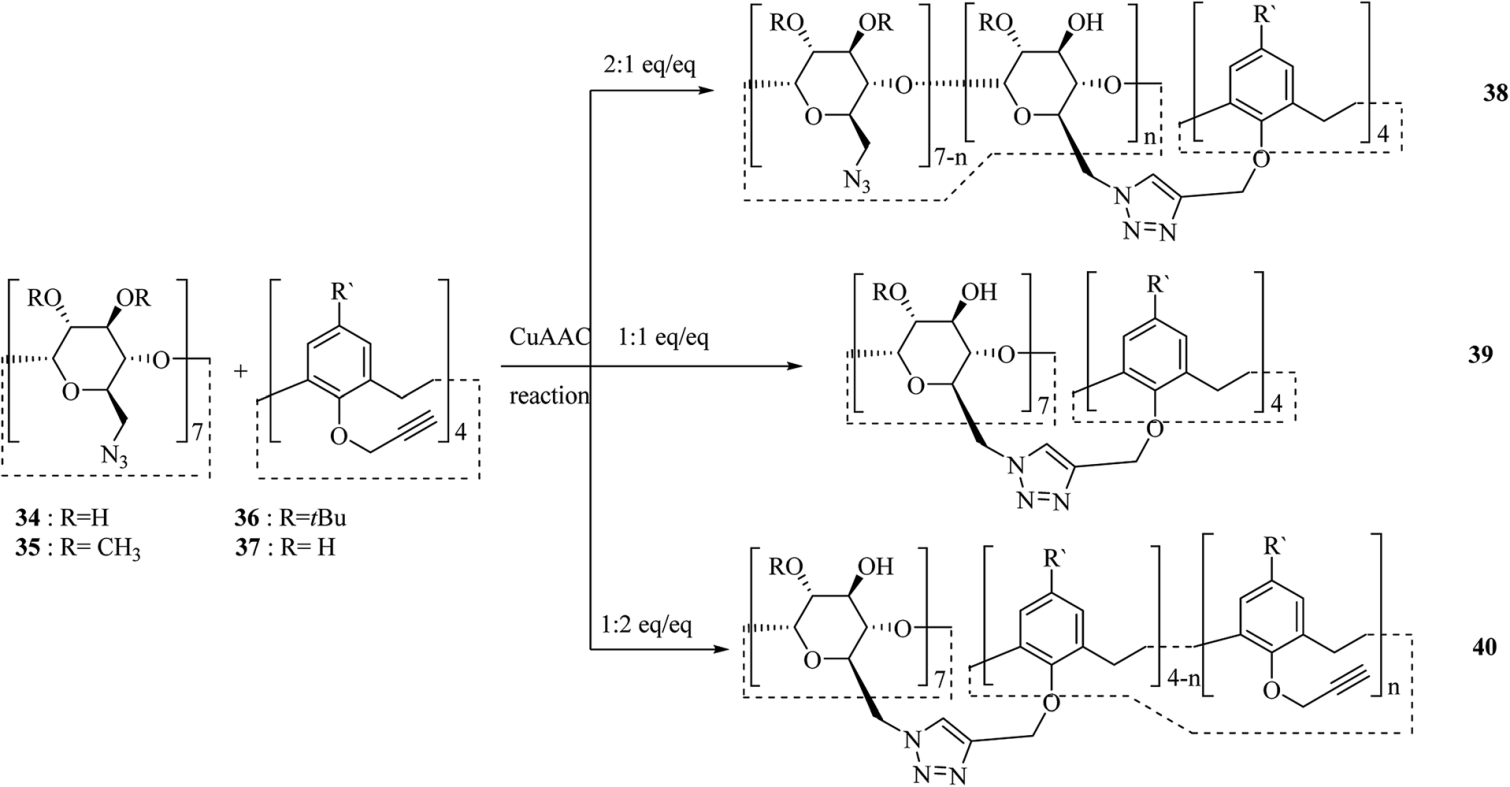
Chemical structure of comonomers and general synthesis of copolymers 38, 39 and 40.

One of the major goals in material chemistry is to achieve tunability of physico-chemical properties and sensitivity to external stimuli. Thus, having an efficient strategy for chemical modification is a key issue. To this end, a novel category of nano sponges as a series of copolymers was developed using cyclodextrin and calixarene units through click chemistry. Scheme 11 illustrates the chemical structure of comonomers 34, 35, 36 and, 37 and general synthesis of copolymers 38, 39 and, 40. Specific organic molecules like nitroarenes and commercial dyes were selected as suitable pollutants to evaluate polymer's absorption performance.^40,41^

**Scheme 12:**
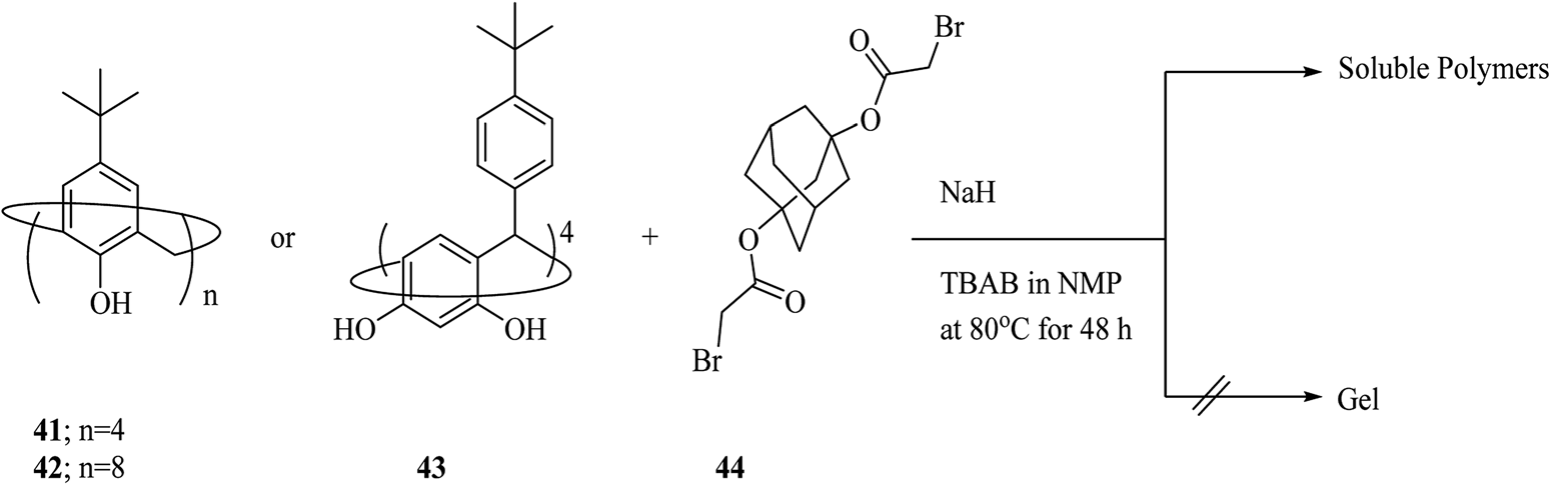
Polymerization reaction of BCA[*n*] (*n* = 4 and 8) and BCRA[4] with ADB.

As another example, Kudo *et al.* investigated the condensation polymerization of *p-tert*-butyl calix[8]arene 42, *p-tert*-butyl calix[4]arene 41, and *p-tert*-butyl calix[4]resorcinarene 43 with 1,3-adamantane dibromo acetate 44 (Scheme 12). As the consequence, they synthesized 3D cross-linking materials with a higher selectivity toward some metal ions in comparison to the calixarene derivatives before polymerization.^42^ The polymers also demonstrated good solubility, desirable film-forming and, thermal stability. Moreover, they investigated the polyaddition reaction of *p-tert*-butyl calix[8]arene 42 and 1,6-hexanediisocyanate 45 in the presence of NEt3 which afforded another soluble copolymer in quantitative yield.^43^

**Scheme 13:**
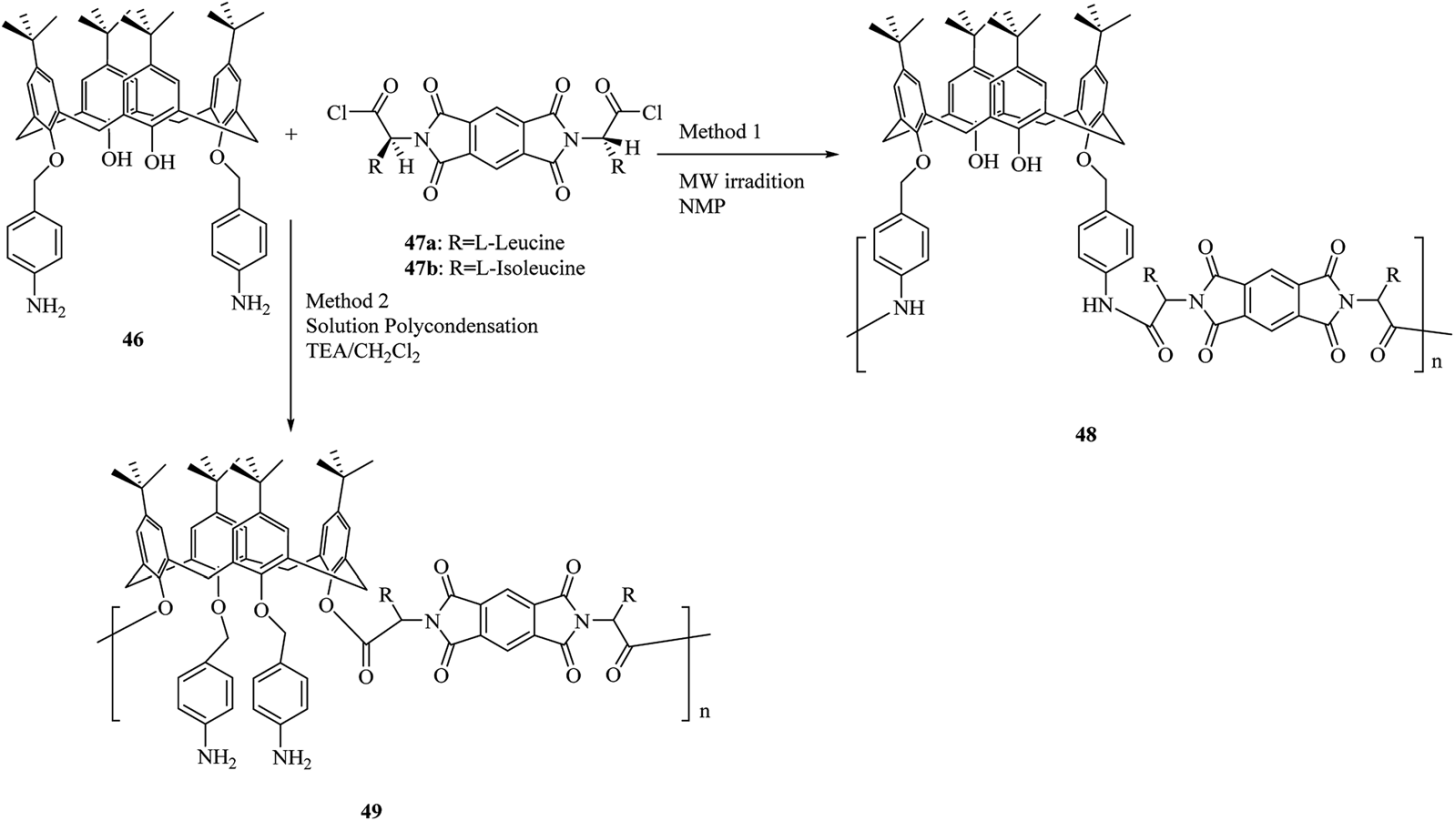
Polycondensation reaction of calix[4]arene diamine 46 with optically active diacid chlorides 47.

As shown by another study, using two diacid chlorides 47 that are optically active, a set of new optically active polymers 48 and 49 containing calix[4]arene moieties was obtained through the polycondensation reaction with calix[4]arene diamine derivative 46 (Scheme 13). Solid–liquid sorption processes were used to study the complexation of the polymers towards specific alkali metals and toxic transition metal cations. As the obtained results showed in comparison to the calix[4]arene starting monomer, the examined polymers are reliable polymeric ionophores for alkali metal cations such as Na^+^, K^+^ and, also for toxic heavy metal cations like Cu^2+^, Co^2+^, Cd^2+^ and, Hg^2+^. In addition, these polymers promise great uses in chiral stationary phases to separate enantiomers in ionic media.^44^

**Scheme 14:**
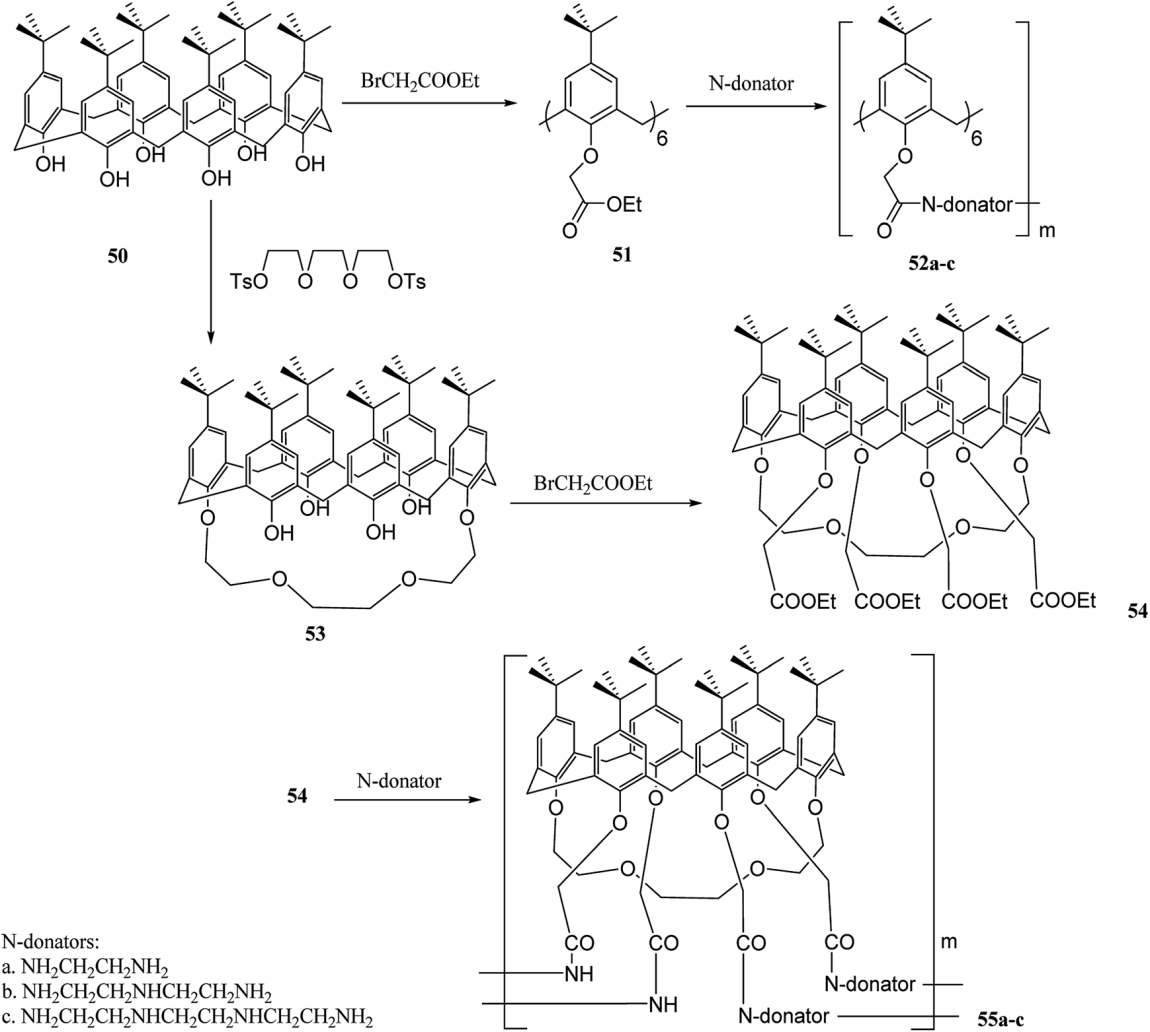
Preparation of polymers 55a–c.

Calixcrowns belong to calixarenes family that demonstrate well-preorganized structures and stronger binding sites compared to calixarenes and crown ethers.^45,46^ Recently, several studies have been developed based on calixcrowns. For instance, Yang *et al.* have investigated calixcrown polymers through condensation of calix[6]arene hexaesters 51 or calix[6]-1,4-crown-4 tetra esters 54 with poly ethylene imine. It is possible to obtain a series of new calix [6]amide-based polymers 52a–c and calix[6]-1,4-crown-4-based polymers 55a–c (Scheme 14). There are also studies on the adsorption properties of these polymers with some cations. The adsorption results for Na^+^, K^+^, Ag^+^, Hg^2+^, Cu^2+^, Co^2+^ and Ni^2+^ are listed in Table 1.^47^

**Table 1:** Adsorption capacities (10^−5^ mol mg^−1^) of polymers 52a–c and 55a–c

	52a	52b	52c	55a	55b	55c
Na^+^	3.2	2.8	0.9	12.1	19.5	18.2
K^+^	4.9	4.5	1.2	10.0	8.1	10.7
Ag^+^	38.5	35.5	32.9	23.3	25.6	25.2
Hg^2+^	31.7	14.3	4.4	7.1	4.9	11.4
Cu^2+^	10.6	10.3	9.3	64.5	60.8	52.6
Co^2+^	23.5	21.3	18.6	58.9	62.7	54.8
Ni^2+^	25.7	18.6	20.5	64.1	70.2	65.3

**Scheme 15:**
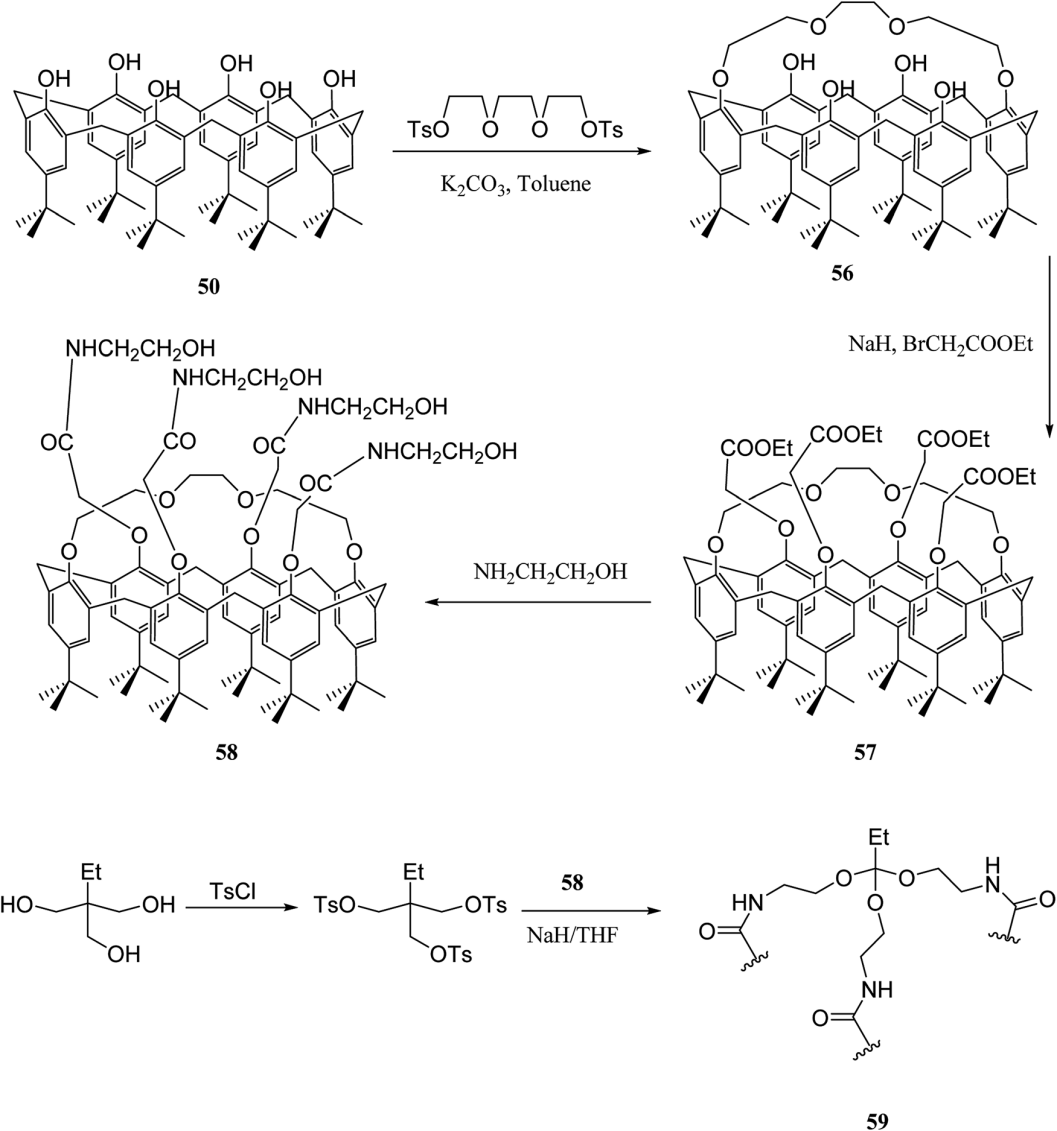
Preparation of polymer 59.

Guo *et al.* developed different types of calix [6]-1,4-crown-based polymers. These novel polymers showed excellent adsorption capacities for different aniline derivatives. The adsorption percentages of these aniline derivatives were higher than 90%. Moreover, there was a good reused property for polymer 59 (Scheme 15).^48^

### 2.3 Molecular recognition by calixarene-based covalent polymers

**Scheme 16:**
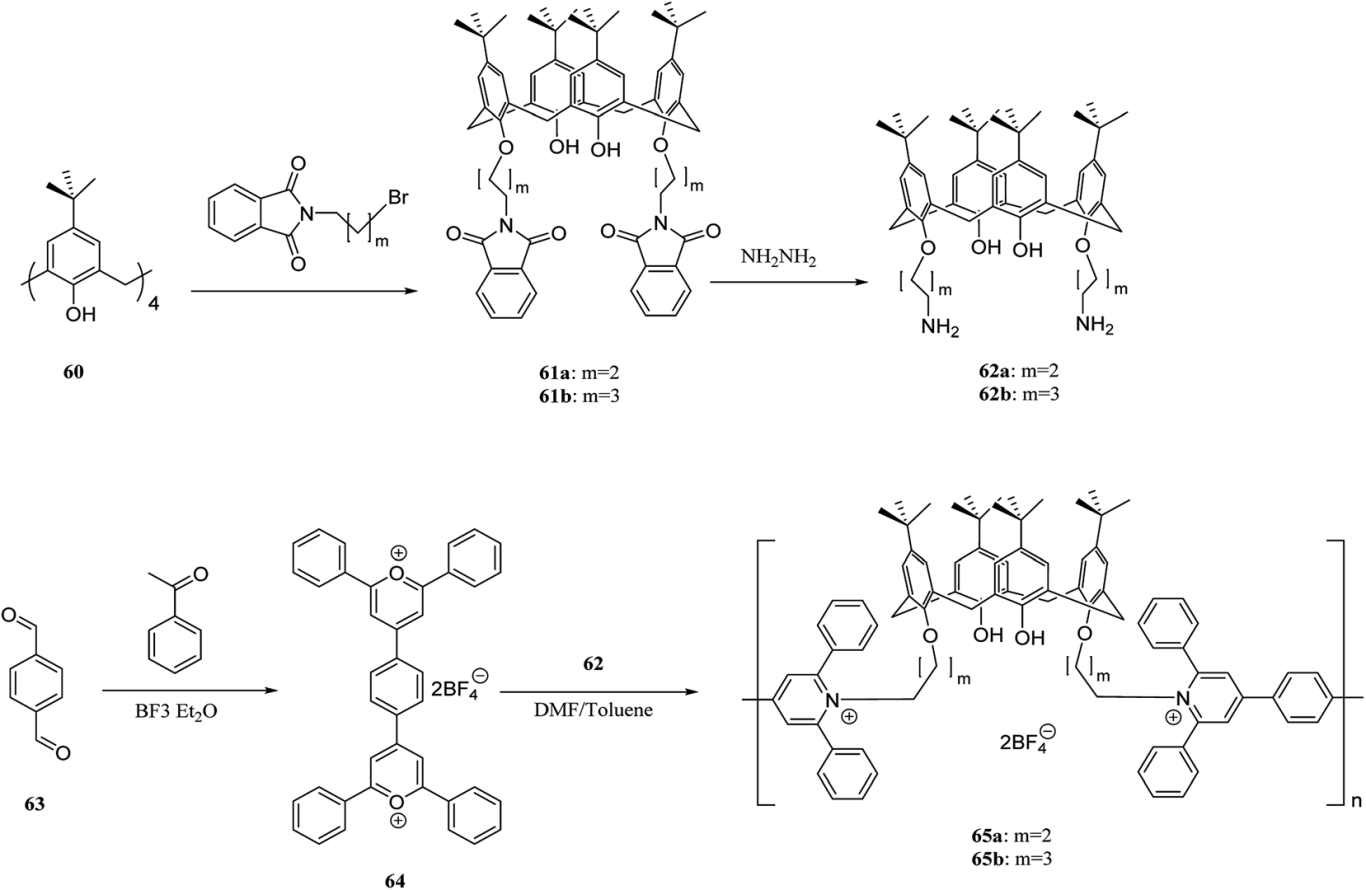
Preparation of poly(pyridinium) 65a–b.

There is considerable interest in developing efficient artificial receptors for molecular recognition and sensing. Calixarene-based covalent polymers have an important role as receptors for the selective recognition of neutral biomolecules and other guest molecules. For example, Lu *et al.* synthesized novel fluorescent calixarene-based polymers using polycondensation of 4,4-(1,4-phenylene)bis(2,6 diphenylpyrylium tetrafluoroborate) 64 with calix[4]arene diamines 62a and 62b to prepare poly(pyridinium) salts 65a and 65b (Scheme 16). The adsorption and photoluminescence spectra of the two polymers were examined using a fluorescent titration method. As the results showed, pseudomonas fluorescent DNA demonstrated a notable interaction with this type of polymers through electrostatic interactions.^49^

**Scheme 17:**
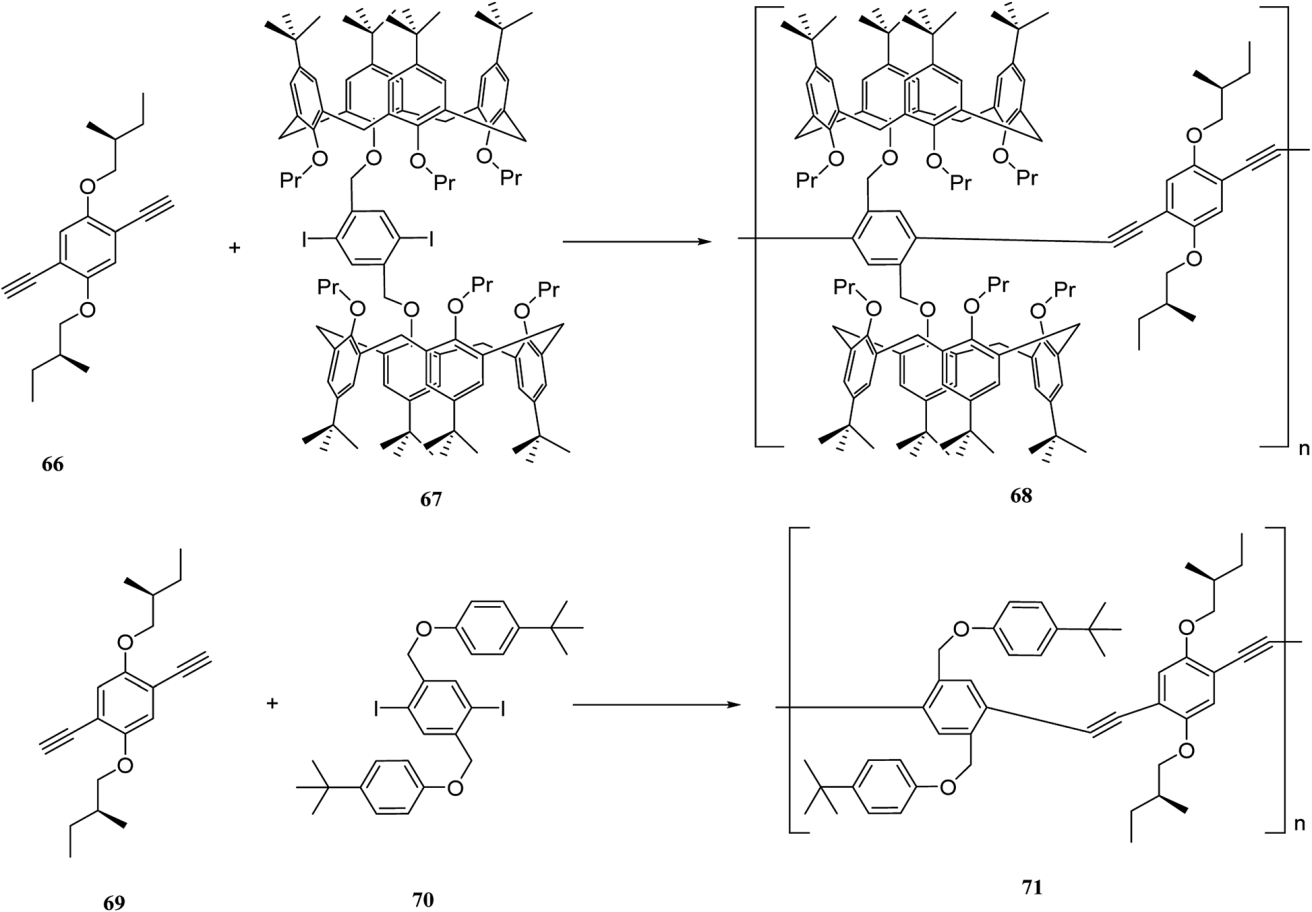
The presentation of chiroptical polymers 68, 71.

The past two decades have witnessed a steady growth of using *p*-phenyleneethynylenes (*p*-PEs). Poly(*p*-PEs) are one of the conjugated macromolecules that demonstrate incredible fluorescence efficiencies. The incorporation of suitable molecular receptors into aryleneethynylene polymer frameworks can create specific supramolecular hosts to interact with certain guests and increase the sensory responses.^50^ Prata *et al.* synthesized a new category of chiral supramolecular polymers using calixarene and aryleneethynylene monomers. The polymers are optically active and including bulky bis-calix[4]arene units and chiral side-chain on the main skeleton. The 68 and 71 are the target polymers that are illustrated in Scheme 17 and, aryleneethynylene is used as a comonomer. In addition, the chiroptical activity and fluorescence of 68 and 71 were studied using different temperature range (−10 °C to 60 °C). Both polymers were examined in terms of enantiomeric recognition abilities towards (*R*)- and (*S*)-α-methyl benzylamine. As the results showed, the calixarene-based polymer 68 has a significant enantiodiscrimination.^51^

**Scheme 18:**
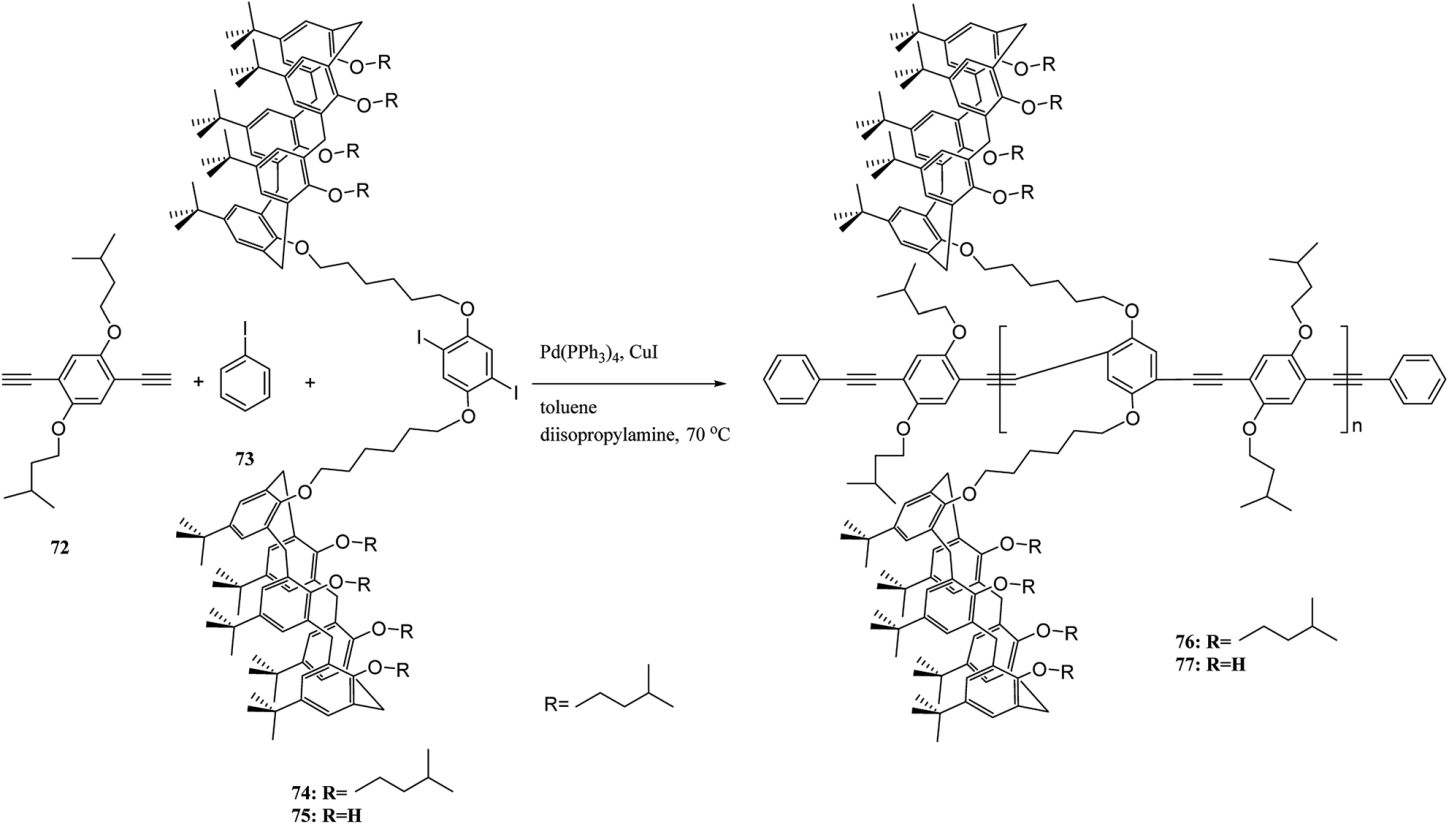
The polymerization of 1,4-diethynyl-2,5-bis(4-methylpentyloxy)benzene 72 with a bis-calix[5]arene 74.

**Scheme 19:**
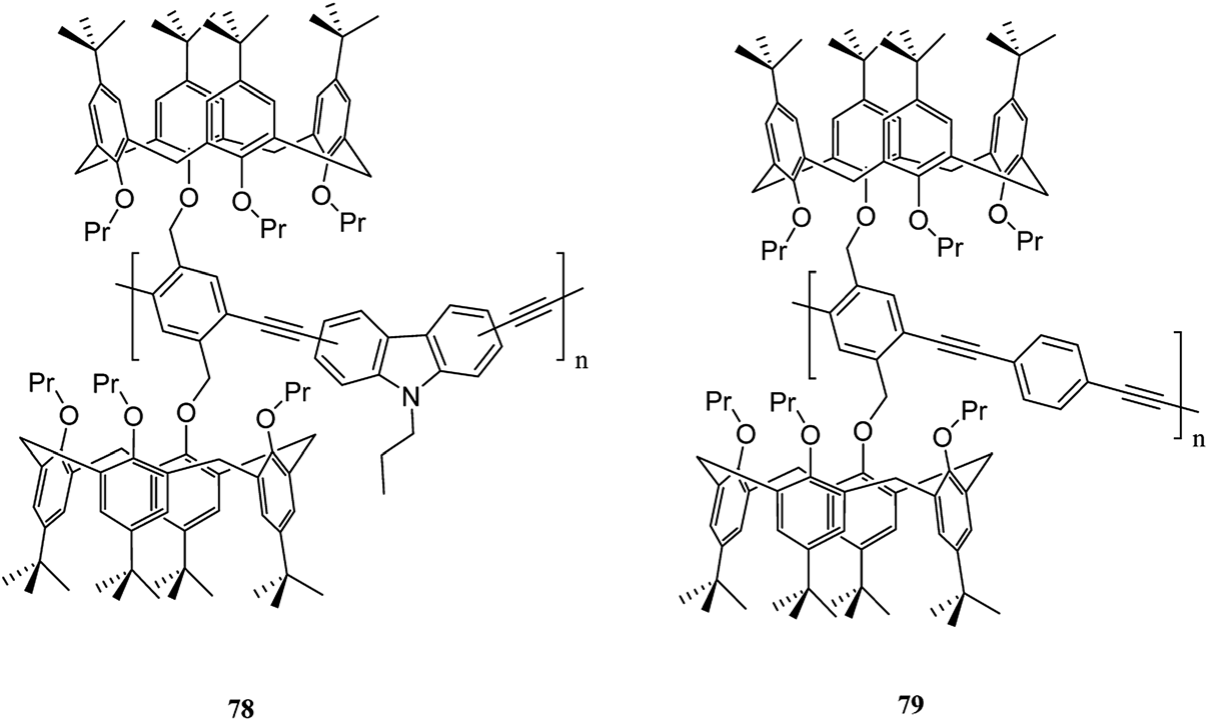
The structures of conjugated polymers 78 and 79.

Moreover, Pappalardo *et al.* indicated that synthesis of the poly(*p*-phenylene ethynylenes) 76 was achieved through the Pd-catalyzed cross-coupling reaction of 1,4-diethynyl-2,5-bis(4-methylpentyloxy)benzene 72 with a proper amount of bis-calix[5]arene 74. Through this, they achieved a polycapsular polymer network assembly.^52^ In addition, Fraix *et al.* developed a poly(*p*-phenylene ethynylenes) polymer 77 with two π-rich cone-like calix[5]arene cavities (assembling cores) connected to a rigid *p*-phenylene ethynylene spacer so that it is possible to assemble the target polymer using complementary C_60_ fulleropyrrolidine guest in solution (Scheme 18).^53^ Barata *et al.* researched to achieve two fluorescent molecular receptors using the conjugated polymers 78 and 79 that are utilized to detect explosive nitro aliphatic compounds such as nitromethane and 2,3-dimethyl-2,3-dinitrobutane. They showed that a thin film of the two polymers 78 and 79 had a notably high sensitivity and selectivity towards these analytes (Scheme 19).^54^

**Scheme 20:**
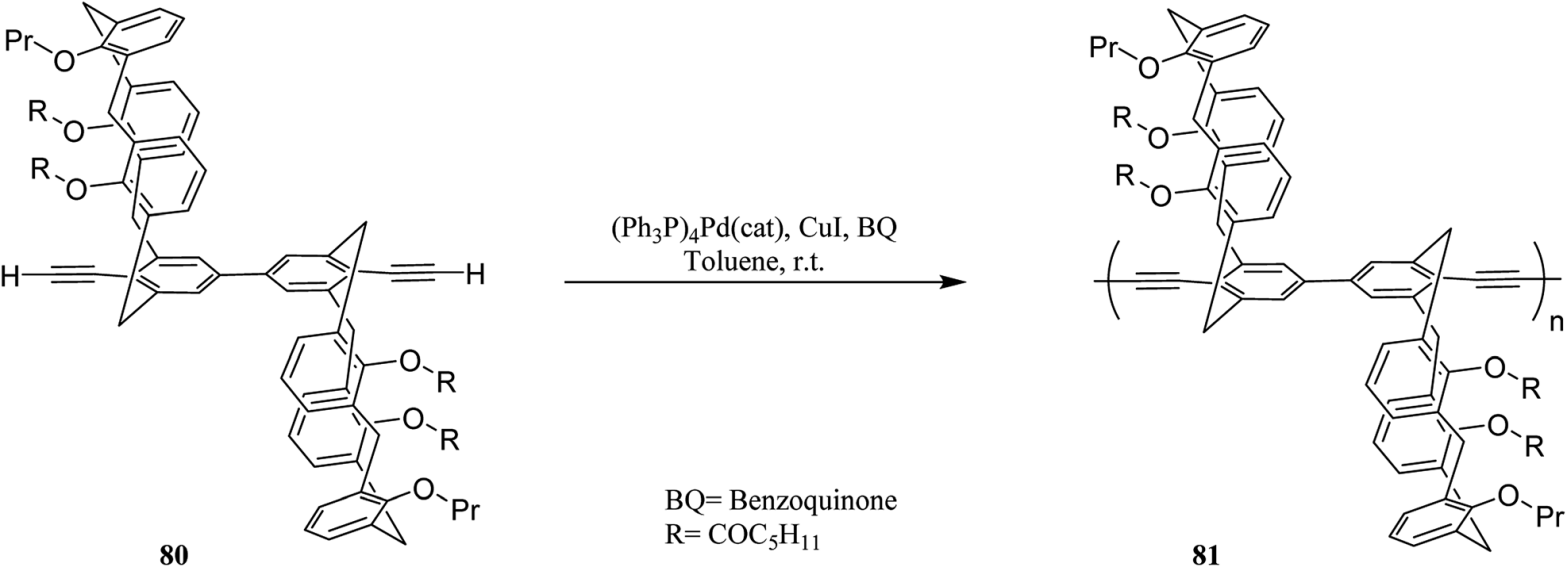
The structures of conjugated polymer 81.

**Scheme 21:**
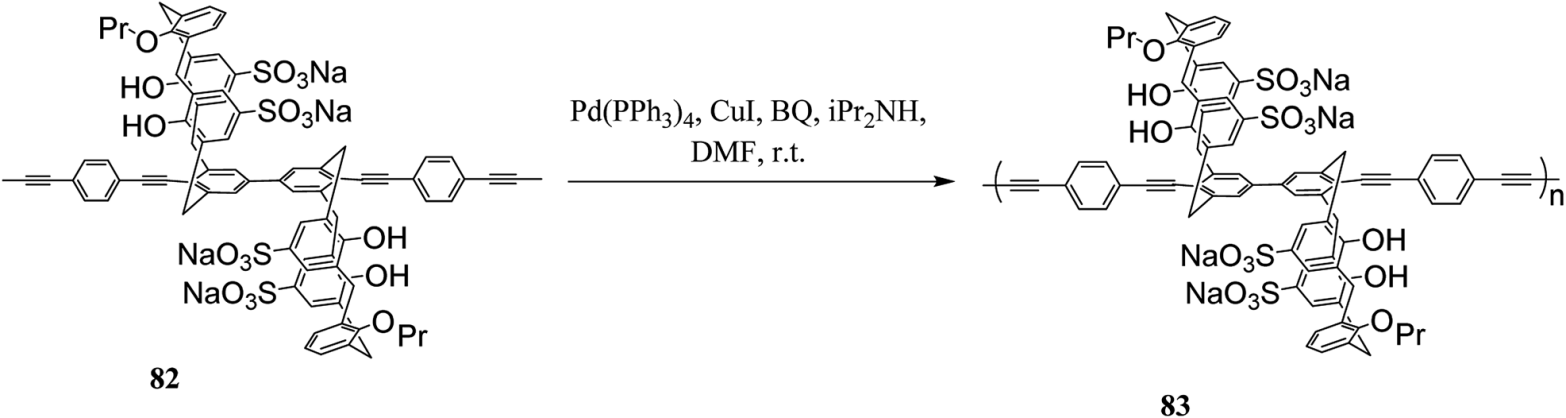
The structures of conjugated polymer 83.

Molad *et al.* reported a fluorescent 5,5-bicalixarene-based polymer 81 that the calixarene units are incorporated in the conjugated polymeric chain (Scheme 20). The conjugated polymer can respond to a small molecule complexation inside the hydrophobic cavity as the first tubular fluorescent polymer with fused calixarene cavities. In particular, it demonstrated a reversible rapid fluorescence quenching upon the interaction of gaseous nitric oxide with the calixarene moiety.^55^ Another example of fluorescent 5,5-bicalixarene-based conjugated polymer 83 was studied by Ahuja *et al.* as a modified water-soluble polymer (Scheme 21). It can respond to gaseous nitric oxide like polymer 81*via* host–guest complexation mechanism. In fact, conjugated polymer bearing 5,5′-bicalixarene scaffolds have demonstrated enhanced sensitivity compared with their monomers due to the molecular wire effect.^56^

### 2.4 Calixarene-based covalent polymers as gels and hydrogels

**Scheme 22:**
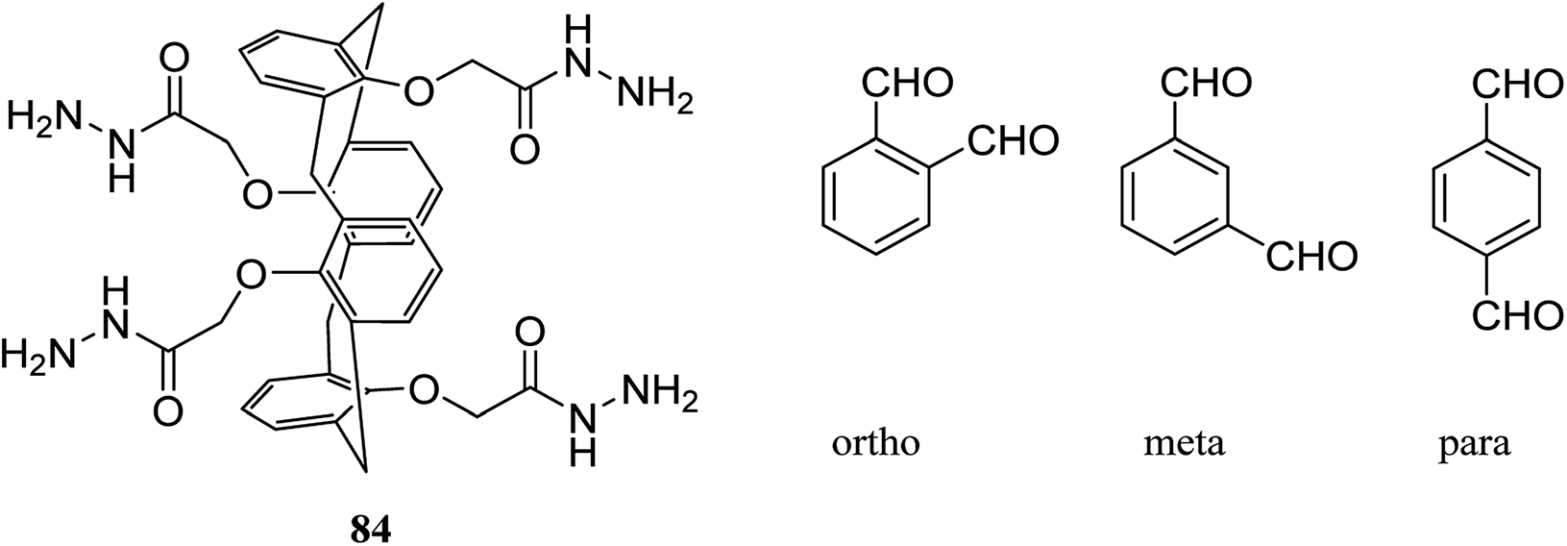
Chemical structure of compound 84.

Gels are soft materials with numerous applications such as controlled release and soft tissue. These materials, including organogels and hydrogels were mostly produced through chemical reactions. Another form of the gels are physical gels that were produced through non-covalent interactions like hydrogen bonding, π–π stacking, electrostatic interaction, and metal coordination.^57^ Lee *et al.* recently studied oligomer-type gels afforded through hydrazone bond formation by using of a calix[4]arene gelator. In fact, calix[4]arene-based compound 84 was used as a gelator containing hydrazine groups for reaction with different aldehyde derivatives such as *ortho*, *meta*, and *para* to produce the gels (Scheme 22). Afterward, the drug delivery property of the obtained gels was evaluated.^58^

**Scheme 23:**
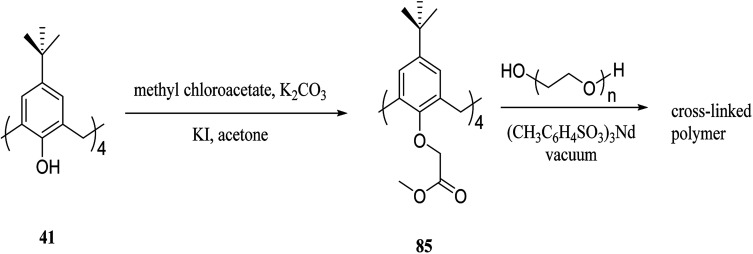
Polycondensation reaction of *p-tert*-butylcalix[4]arene derivative 85 and di hydroxyl poly(ethylene glycol).

**Fig. 1:**
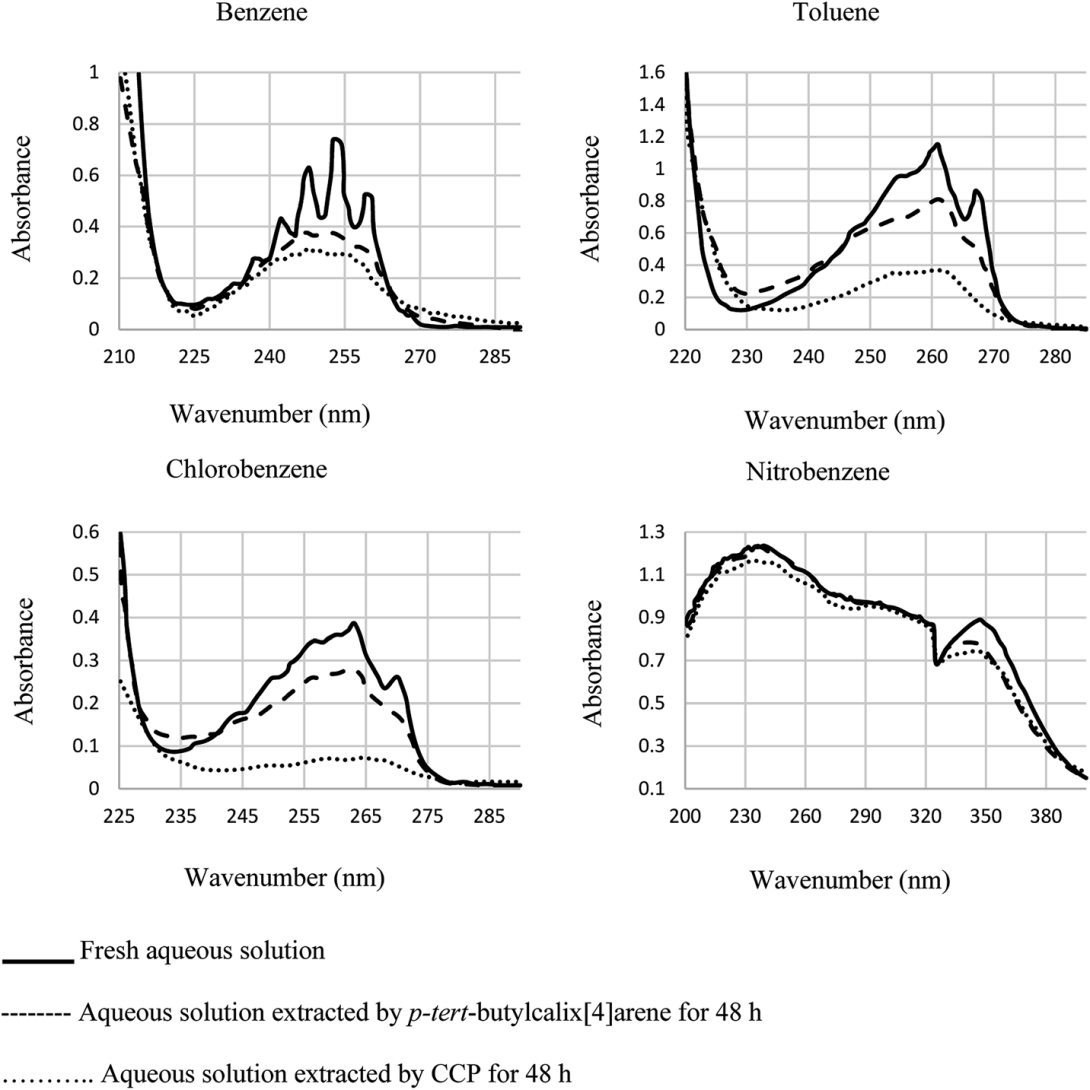
The UV spectra of the aromatic aqueous solutions before and after the extraction by CCP hydrogel.

**Fig. 2:**
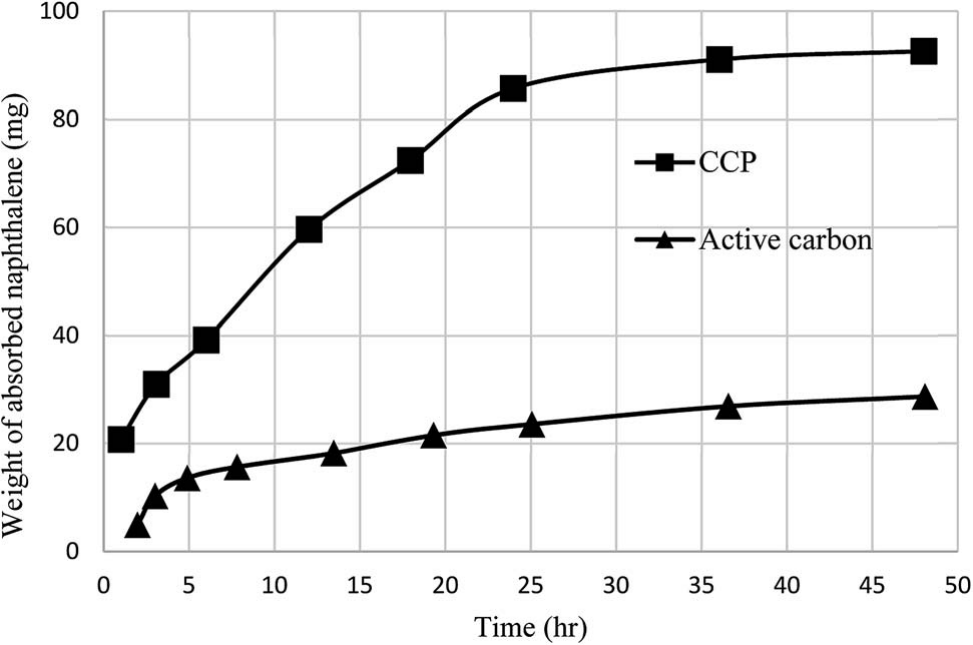
Competitive diagram between CCP and active carbon for naphthalene adsorption from gas phases.

As another work, a new calix[4]arene-poly(ethylene glycol) cross-linked polymer (CCP) was synthesized using a polycondensation reaction between *p-tert*-butylcalix[4]arene derivative 85 and di hydroxyl capped poly(ethylene glycol) catalyzed by neodymium tosylate (Scheme 23). The obtained hydrogel contained 66.9% water and 33.1% CCP, which is capable to selectively extract aromatic organic molecules out of aqueous solution based on the diameter of the guest molecules. The UV spectra of the aromatic aqueous before and after the extraction by CCP hydrogel is illustrated in Fig. 1. In terms of extracting toluene and chlorobenzene molecules out of aqueous solution, CCP hydrogel can extract them better than benzene. However, it is almost incapable of extracting nitrobenzene (Table 2). Additionally, as illustrated in Fig. 2, CCP adsorbs naphthalene from the gas phase and demonstrates notably superior capacity comparing with active carbon. This promises potential uses in the area of removing pollutants.^59^

**Table 2:** Extraction of aromatic molecules from aqueous solution

Run	Guest molecule	Maximum UV absorbance	Diameter (Å)	*C*_initial_ (ppm)	*C*_final_ (ppm)	*C*_hydrogel_ (ppm)
1	Nitrobenzene	241 nm, 1.234	5.59	700	657	717
2	Toluene	261 nm, 1.157	5.41	700	221	7983
3	Chlorobenzene	263 nm, 0.386	5.39	700	128	9533
4	Benzene	253 nm, 0.737	5.01	550	223	3783

### 2.5 Other studies related to calixarene-based covalent polymers

**Scheme 24:**
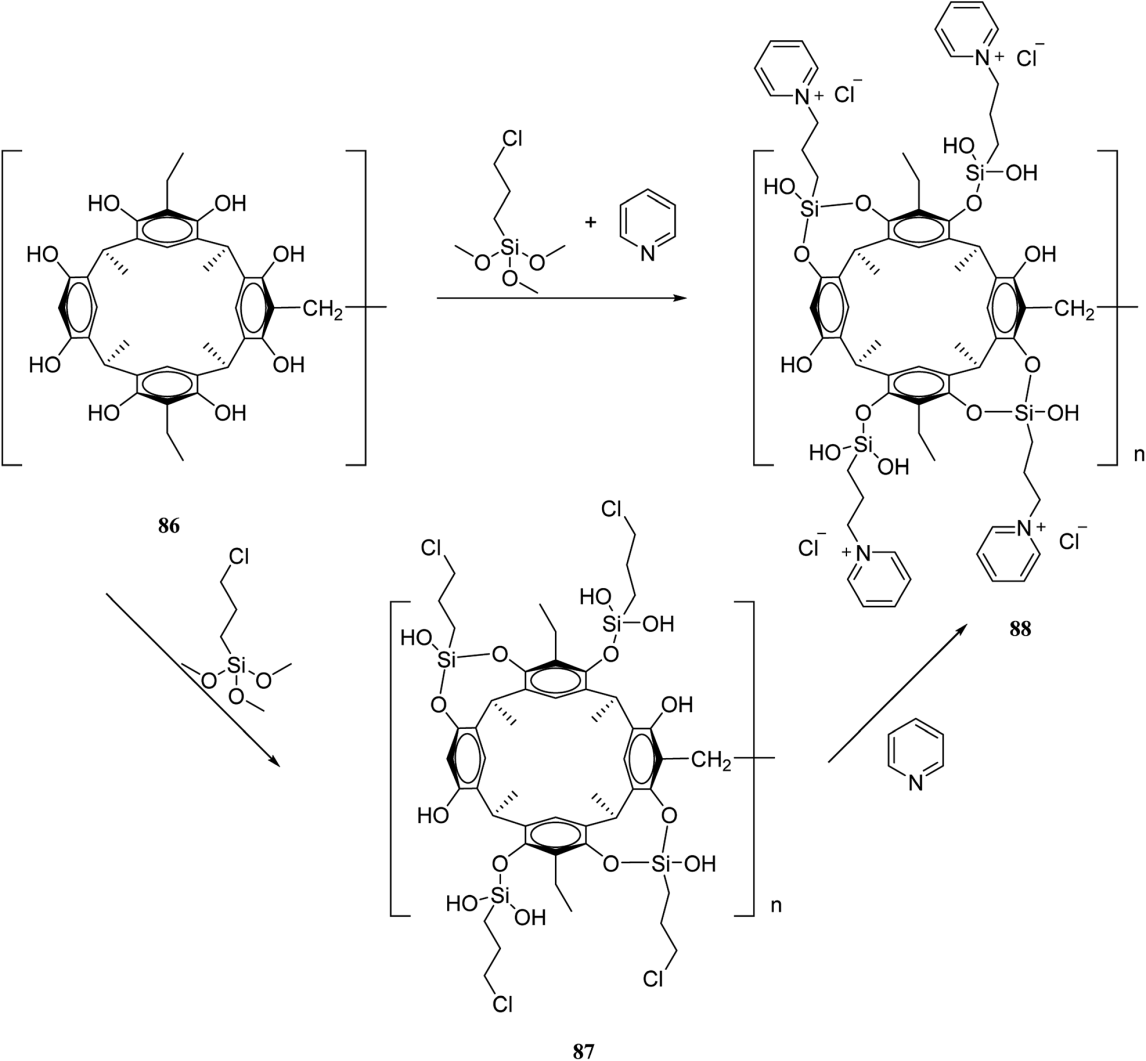
Synthesis route of the cationic polymer 88.

**Scheme 25:**
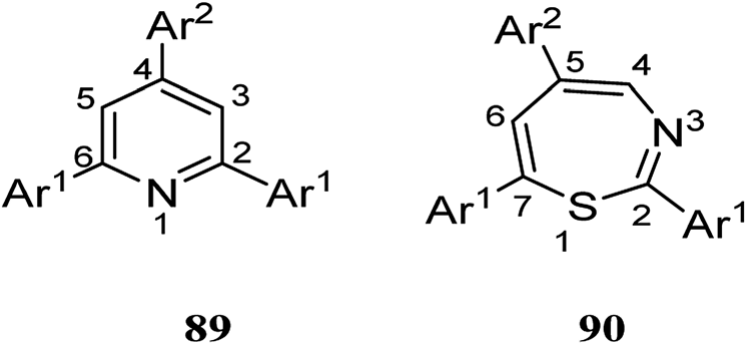
Chemical structure of compound 89 and 90.

The recent two decades have witnessed the main problem in the field of organic synthetic chemistry, including the absence of an environment friendly process.^60^ In response to such concerns, various strategies in modern chemistry have been exploited. For instance, the advantages of polymer-supported reagents have been proven to accommodate the principles of green chemistry in the synthesis of desired products. Mouradzadegun *et al.* suggested calixarene-based cationic polymer 88 with pendant pyridinium groups using calix[4]resorcinarene 86 (Scheme 24). This functionalized cationic polymer can act as a passive reagent for a pure and efficient synthesis of bioactive 2,4,6-triarylpyridine 89 and novel 2,5,7-triaryl-1,3-thiazepine 90, which are the key moieties in different biologically active molecules (Scheme 25). In this study, a porous polymer has been used as the carrier to carry out a highly selective and mild chemical reaction.^61^

**Scheme 26:**
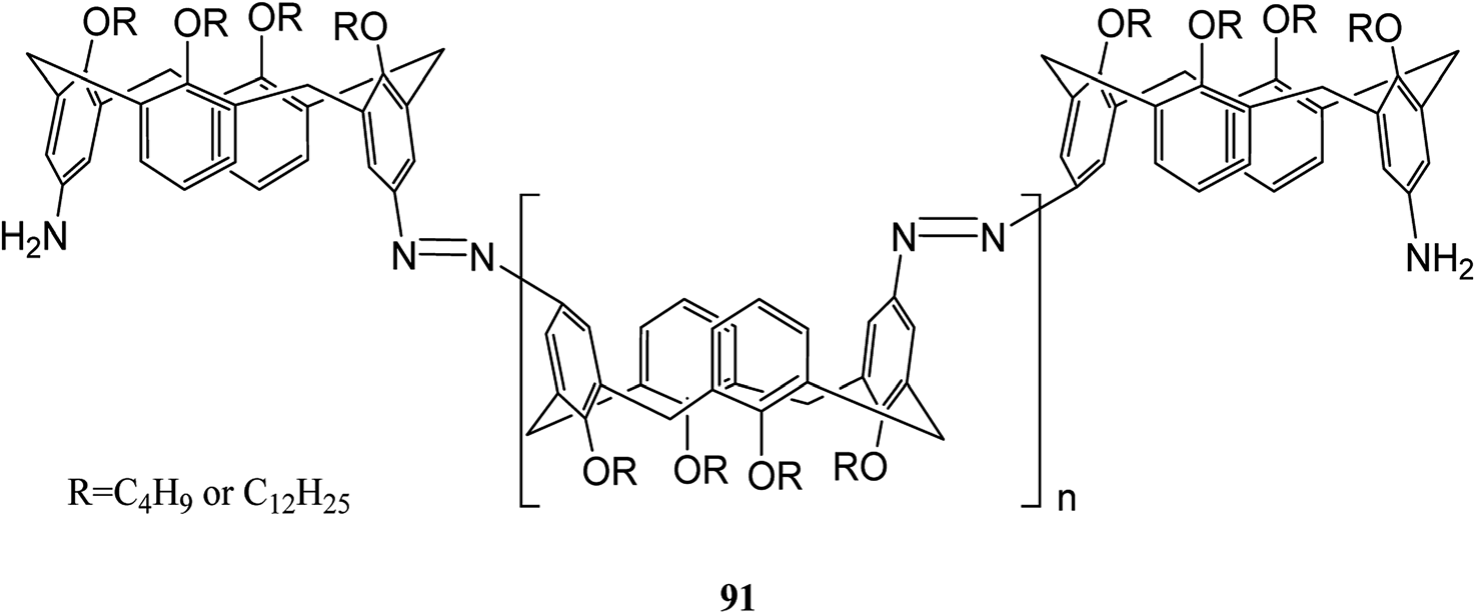
The chemical structure of polymer 91.

Wiktorowicz *et al.* studied the photo-isomerization behavior of poly(azo calix[4]arene) 91 using the different lengths of lower rim substituents (*n*-butyl and *n*-dodecyl) (Scheme 26). They also examined the complex formation properties using a pyridinium-based compound as a low molar mass guest. The irradiation caused some changes in the cone conformation of the calix[4]arene units which had an influence on the host–guest interactions of the polymer so that the interactions decreased as the polymer was in the *cis*-rich state.^62^

**Scheme 27:**
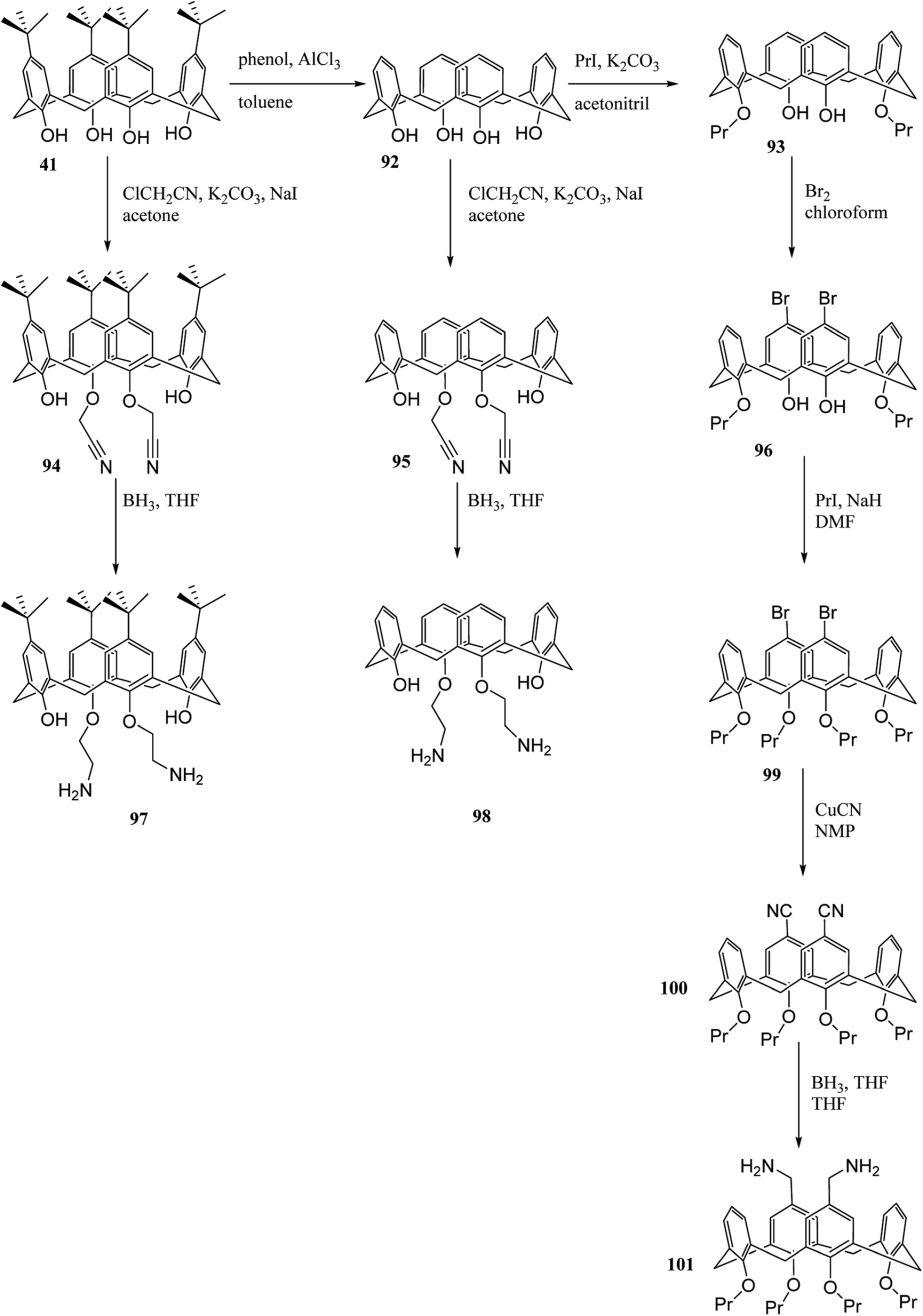
The synthesis route of diamino calix[4]arene derivatives as monomers.

Aromatic polyimides (PIs) have many particular chemical and physical properties. Various applications from PIs have been found in microelectronics, airplanes, aerospace exploration, and polymeric separation membranes. Hobzova *et al.* studied the synthesis and characterization of calix[4]arene-containing polyimides. Scheme 27 illustrates the synthesis of diamino calix[4]arene derivatives 97, 98 and, 101 (DACXA) as a macromonomers. The calix[4]arene-containing polyimides are yielded by the reaction between DACXA and phthalic anhydride. The thermal and dynamic mechanical behavior, wide-angle X-ray diffraction and solubility of the resulting polyimide films in selected solvents were examined.^63^

**Scheme 28:**
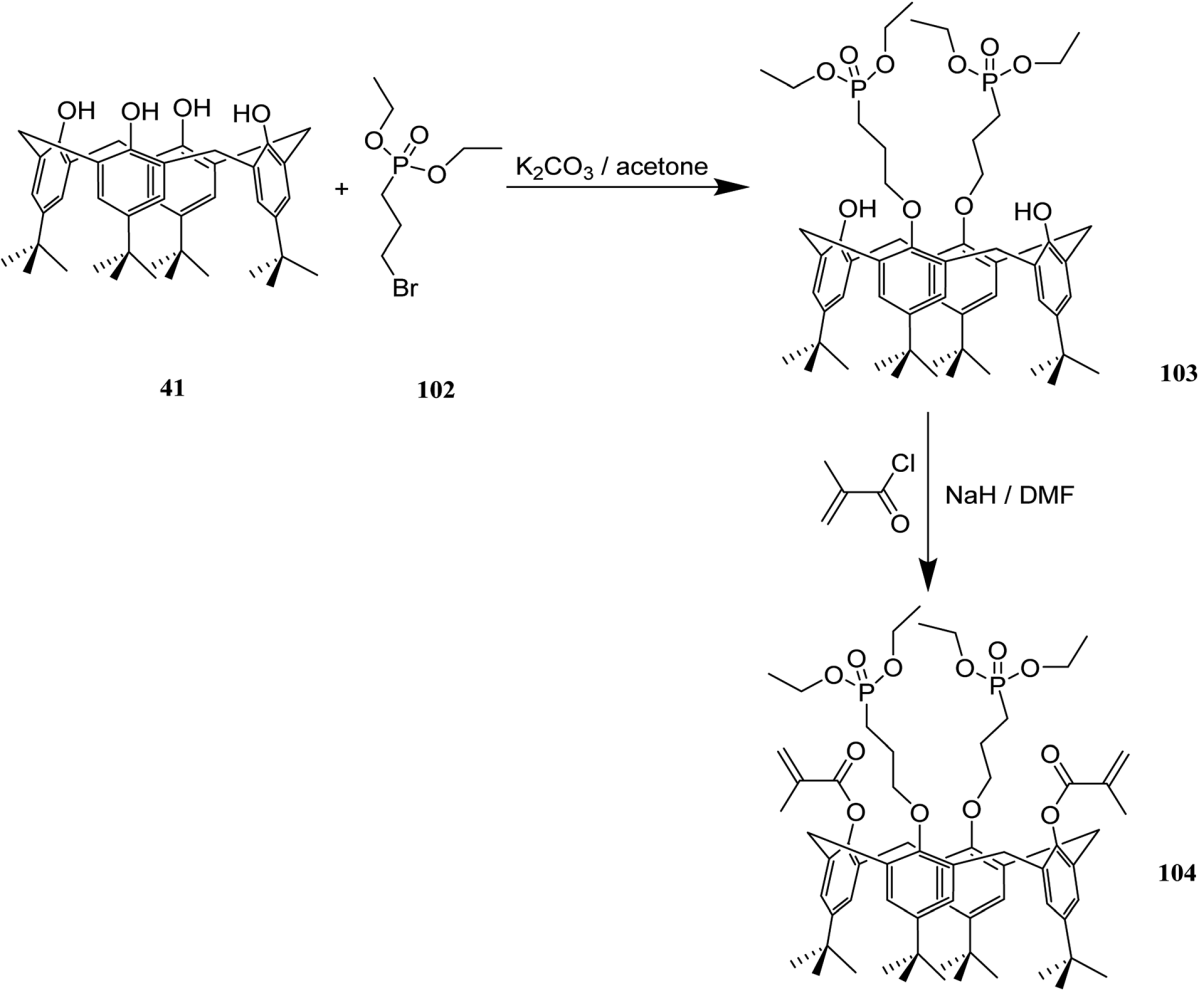
The presentation of methacrylated calix[4]arene phosphonic acids and rout of synthesis.

The application of dental composites for tooth restoration is limited by the bonding strength of composite and dentin. These bonding strengths are affected by the shrinkage during polymerization and the mechanical behavior of the composite. Composite adhesives have been improved by self-etching strongly acidic monomers, monofunctional comonomers and, crosslinking monomers. Due to the fact that there are several possibilities for modification of calix[4]arenes, they are potential candidates to build an etching and crosslinking monomer in one compound. In 2012, methacrylated calix[4]arene phosphonic acids 104 as a functionalized crosslinker for radical copolymerization was studied by Garska *et al.* The adhesive properties of composites containing these calix[4]arene derivatives were investigated as well. The chemical structures and the synthesis route of this methacrylated calix[4]arene phosphonic acids is presented in Scheme 28.^64,65^

## 3. Functionalized polymers *via* calixarene moieties

This section investigates the studies conducted on the polymers which have been functionalized by calixarene motifs. To achieve polymers contained calixarene moieties, calixarene derivatives were immobilized on polymers and resins.^66^ The final products obtained in this manner are counted as the calixarene-functionalized polymers.^67,68^ Recently, different calixarene-based polymers with the incredible shape of cavities have been prepared using upper and lower rim functionalization and the polymers have focused on controlling the environment pollution, metal ions sorption,^69–73^ amino acid separation and *etc.*^74^ In next sections, polymers and resins functionalized *via* calixarene moieties are reviewed.

### 3.1 Calixarene-functionalized Wang resin

**Scheme 29:**
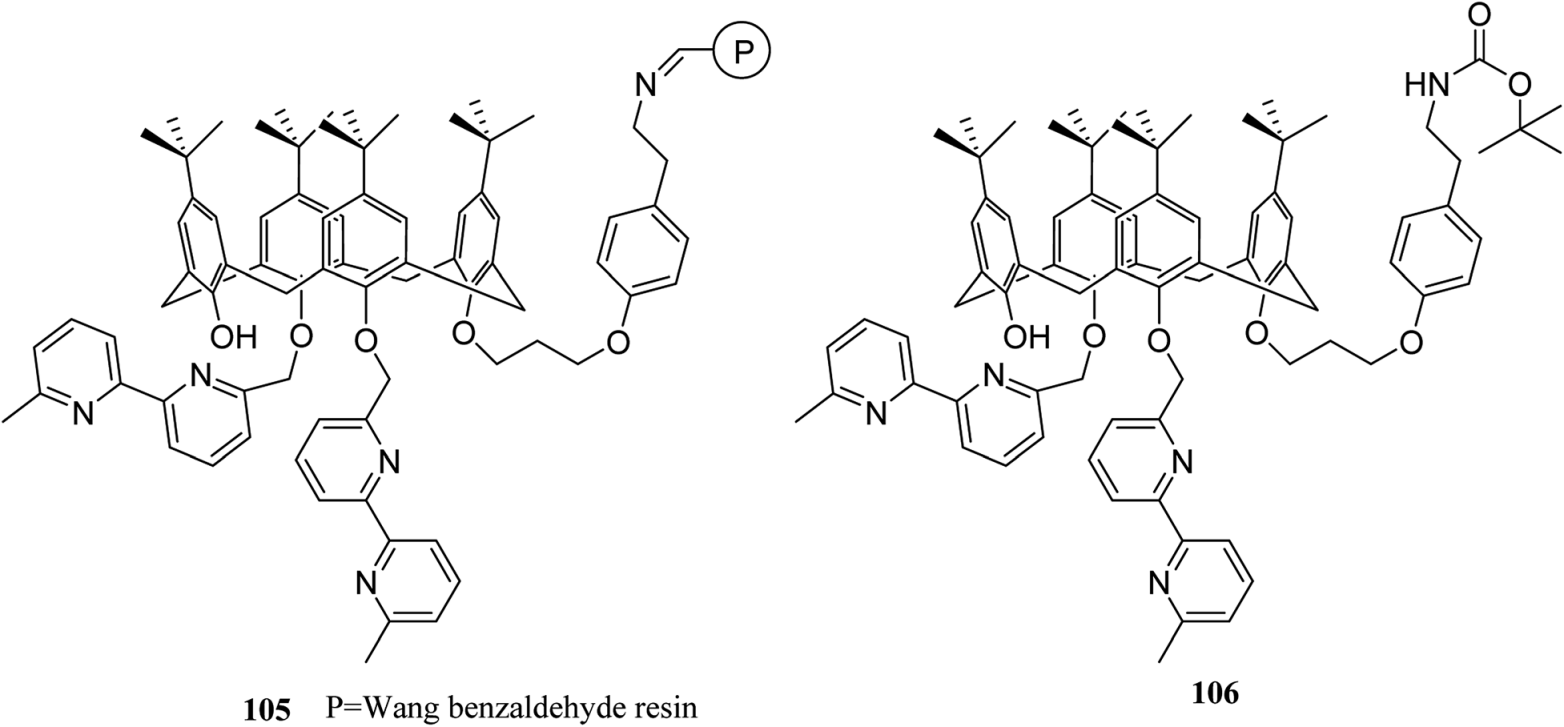
Wang benzaldehyde resin supported calixarene–bipyridyl ligand.

A key feature in calixarene-functionalized polymers is metal complexation that is suitable for extracting and separating studies. The selective extraction of silver(i) and zinc(ii) species include ternary mixtures including, lead(ii), as the key element, was utilized using a Wang benzaldehyde resin supported by calixarene–bipyridyl ligand 105 (Scheme 29). To assess and measure the extraction efficiency and selectivity was done using complexation experiments and ionic liquid chromatography. In addition, the extraction of silver and zinc was done while lead remained in the solution.^75^ Previously, calix[4]arene-functionalized Wang benzaldehyde resin 105 studied by Gaetano *et al.* as a chelating material which displayed a considerable complexation ability towards Cu(i) and Zn(ii) cations.^76^

**Scheme 30:**
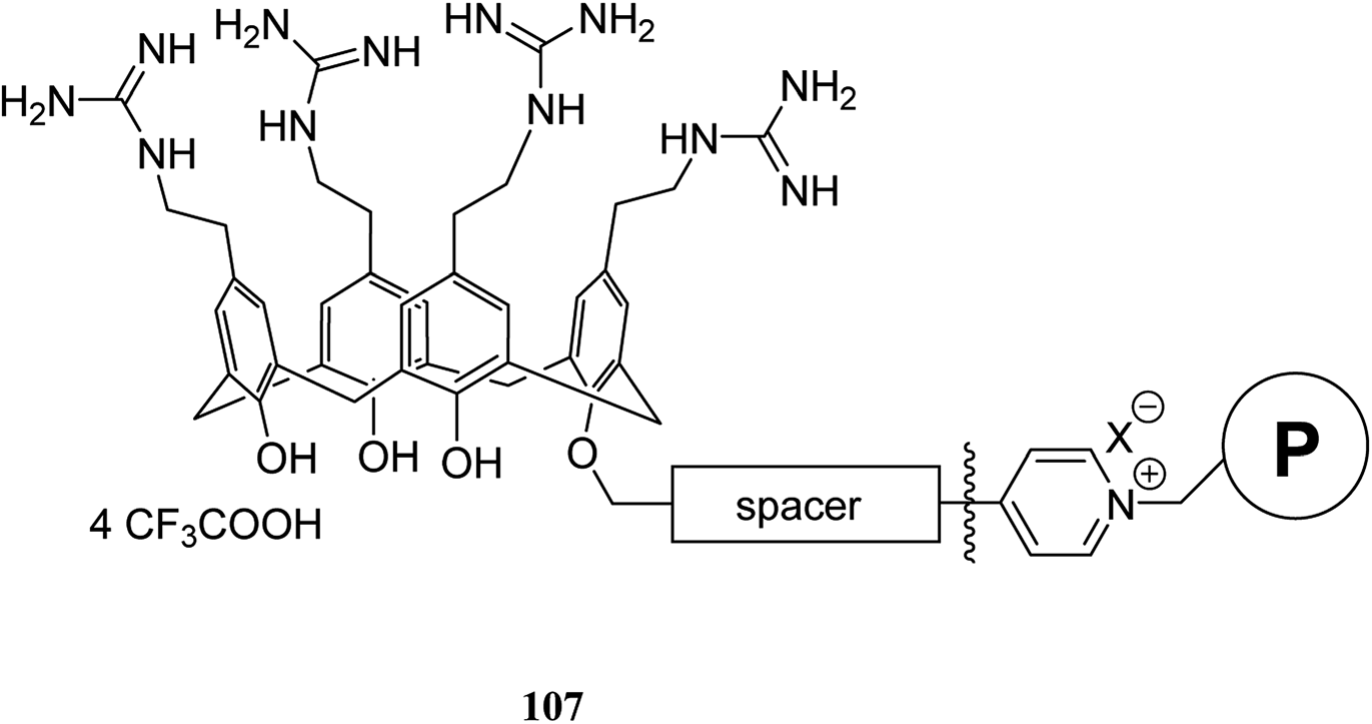
The target calixarene polymer 107.

Lemée *et al.* also anchored the antibacterial tetracationic tetra-*p*-guanidinoethylcalix[4]arene through bottom-up or top-down pathways to the surface of Merrifield and Wang benzaldehyde resins. The attachment involved either a pyridinium linker or an imine/amine one. The sequestering properties of the calixarene-functionalized resins 107 were evaluated towards *Escherichia coli* as a bacterial model by capillary electrophoresis (CE) and the obtained results approved their efficiencies and bacteriophilic behaviors (Scheme 30).^77^

### 3.2 Calixarene-functionalized Merrifield resin

**Scheme 31:**
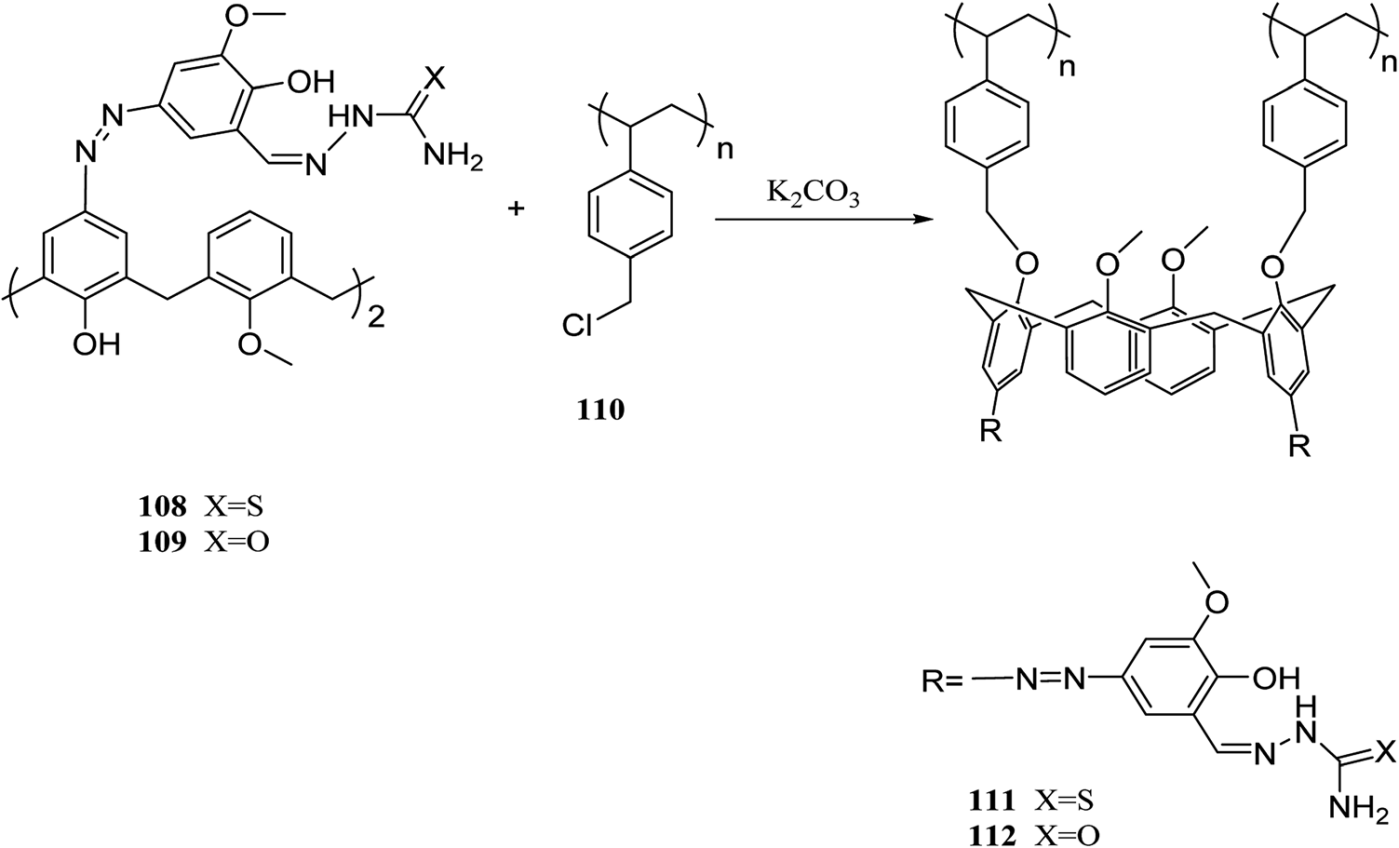
The synthesize route for calixarene-based Merrifield resin 111, 112.

Several studies have been done on extracting metal cations using calixarene-based Merrifield resins.^78–80^ For instance, calix[4]arene-based polymer 111 was synthesized by Jain *et al.* using covalently linking of calix[4]arene-*o*-vanillin thiosemicarbazone 108 through the lower rim into Merrifield resin 110. There has been reports of successful use of resin 111 to separate and trace determine Cr(vi), As(iii), and Tl(i) from natural water samples.^81^ In another study, they also synthesized the functionalized calix[4]arene-ovanillinsemicarbazone 109, which covalently linked to Merrifield resin. The obtained material 112 showed a superior binding affinity towards U(vi) and Th(iv) (Scheme 31).^82^

**Scheme 32:**
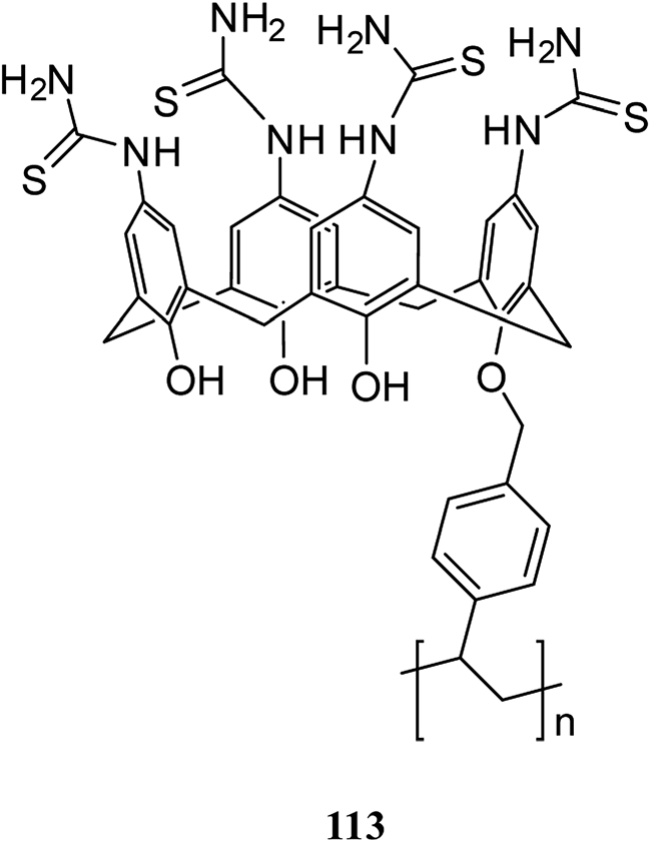
The structure of calix[4]arene-based resin 113.

**Fig. 3:**
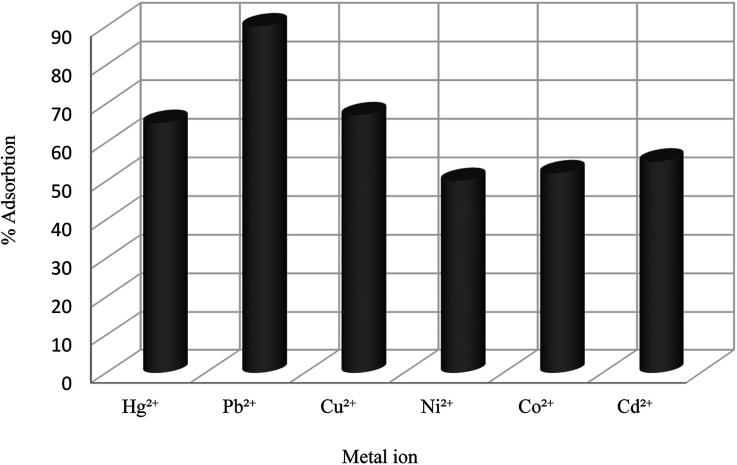
The percent adsorption for metal ions by modified resin 113.

In another study, Bhatti *et al.* examined the adsorption of Pb^2+^ by using the calix[4]arene-based resin 113 as a potential treatment to decrease the danger of environmental water pollution by cationic lead and the different parameters like dosage, concentration, and temperature were improved (Scheme 32). Moreover, solid–liquid extraction experiments with different transition metal picrates like Hg^2+^, Pb^2+^, Cu^2+^, Co^2+^, Cd^2+^ and Ni^2+^ from aqueous media were conducted to study the efficiency of the modified resin (Fig. 3).^83^

**Scheme 33:**
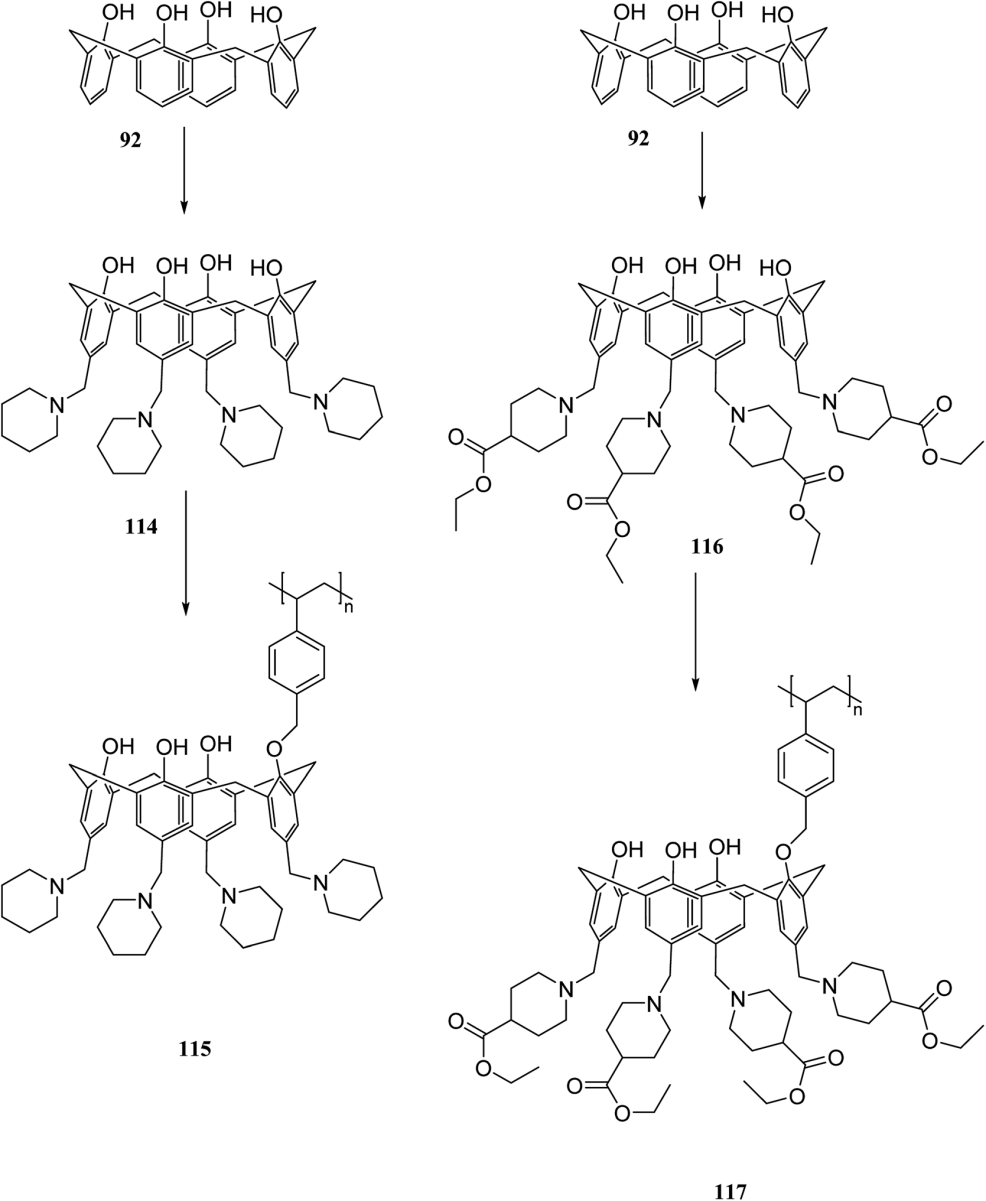
The synthesis route of calix[4]arene-based Merrifield resins 115, 117.

**Fig. 4:**
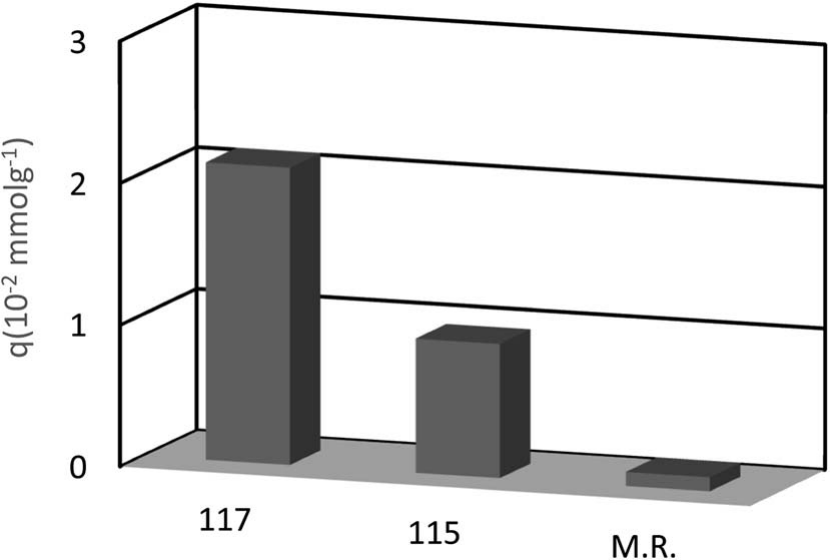
Adsorption capacity of compound 115, 117 and Merrifield resin for dichromate.

Chromate and dichromate anions have drawn a great deal of attention because of their high toxicity and contamination in soils and waters. The extraction of these metal ions is a tedious process as they are associated with a variety of complex species. Various calixarene derivatives have been recently studied as these cavitands have been found useful in the complexation studies.^84^ Akceylan *et al.* synthesized two novel upper rim-substituted calix[4]arene-based Merrifield resins 115 and 117 (Scheme 33). They used liquid–liquid extraction and solid–liquid adsorption processes to evaluate the extraction abilities of monomers and the corresponding polymers toward dichromate. Fig. 4 illustrates the dichromate adsorption capacity of modified resins 115, 117 and Merrifield resin.^85^

### 3.3 Calixarene-functionalized TentaGel support

**Scheme 34:**
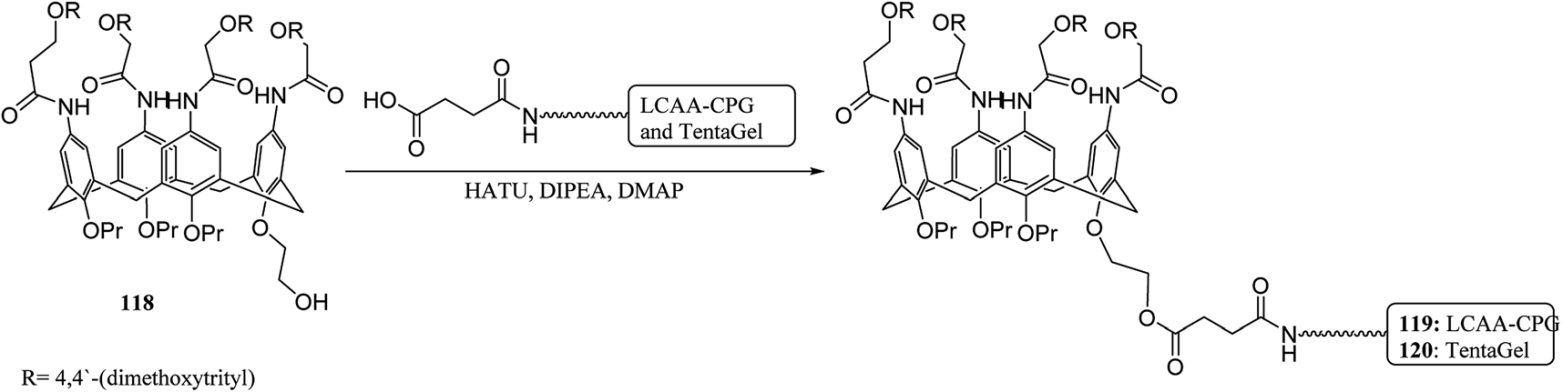
Pore glass and TentaGel supported calixarenes 119, 120.

**Scheme 35:**
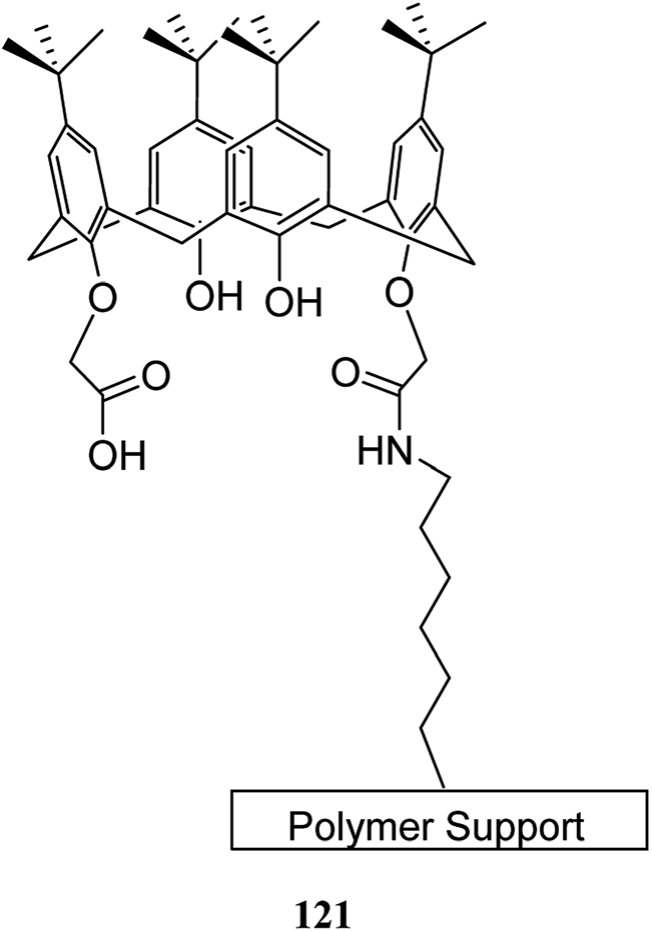
TentaGel supported calixarene 121.

The solid-phase synthesis has notable advantages in organic chemistry like simple separation and purification of materials or good possibility to control organic reactions. Currently, the solid phase synthesis is the key feature in the synthesis of organic materials with repetitive units. Using an easily cleavable ester bond and dimethoxytrityl groups, a calix[4]arene derivative was anchored to controlled pore glass and TentaGel supports. The first example of tetra nucleotide-calixarene which was functionalized at the upper rim was used to show the efficiency of the polymer-supported calixarenes (119 and 120) in solid-phase synthesis (Scheme 34).^86^ Hall *et al.* also studied the application of calix[4]arene modified TentaGel supports 121 for metal cation separation which revealed an excellent extraction of uranium and moderate extraction of cadmium (Scheme 35). In this study, the synthesis of the calixarene modified TentaGel resin was significantly improved with two fewer steps than the standard route. Moreover, the requirement of toxic reagents and solvents was decreased notably.^60^

### 3.4 Calixarene-functionalized silica gel

**Scheme 36:**
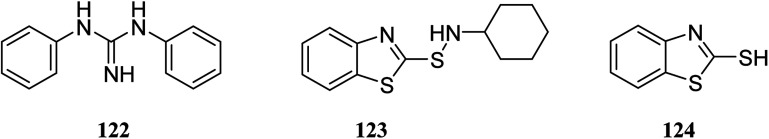
The structure of rubber chemical additives.

**Scheme 37:**
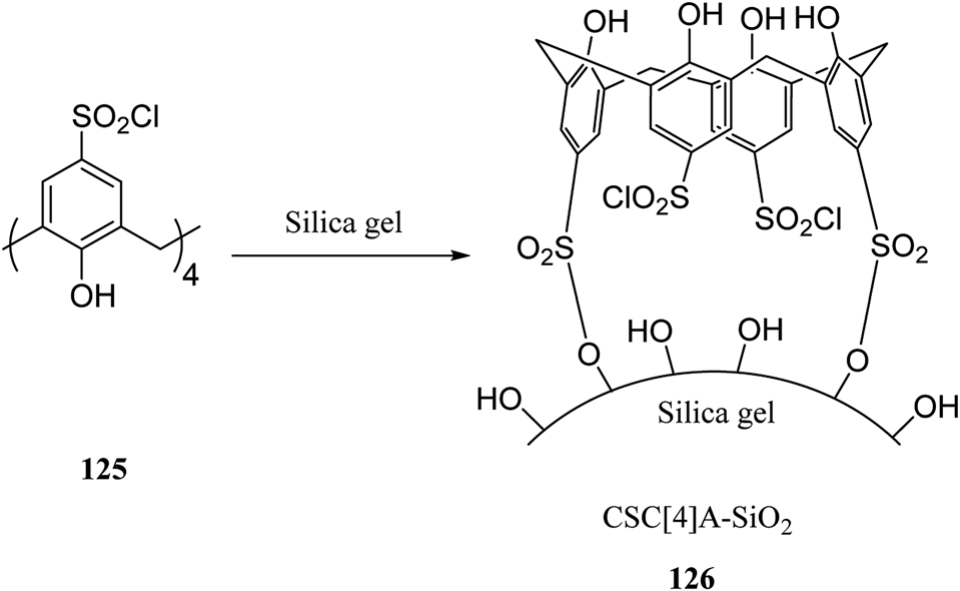
The synthetic strategy for silica bonded calix[4]arene derivative.

There are several key applications for silica gel as the solid support.^87,88^ There have been many studies on the adsorption performance of chemically bonded calixarene to silica gel.^89–92^ For instance, *p-tert*-butyl calix[4]arene was used to modify the surface of silica resin by Temel *et al.*^93^ The yielded sorbent can efficiently and selectively bind the azo dyes^94^ and metal ions.^95,96^ The calixarene-based stationary phases also improved the separation process given the differences in their interactions.^97–101^ For instance, Taghvaei-Ganjali *et al.* reported the adsorption performance of chlorosulfonyl calix[4]arene 125 attached to silica gel for some of rubber chemical additives such as diphenylguanidine 122, *N*-cyclohexyl-2-benzothiazolesulfonamide 123 and 2-mercaptobenzothiazole 124 (Scheme 36). As shown by the results, chlorosulfonyl calix[4]arene-SiO_2_ is a selective sorbent for 122 and 123. Scheme 37 illustrates the synthetic strategy for silica bonded calix[4]arene 126.^102,103^

**Scheme 38:**
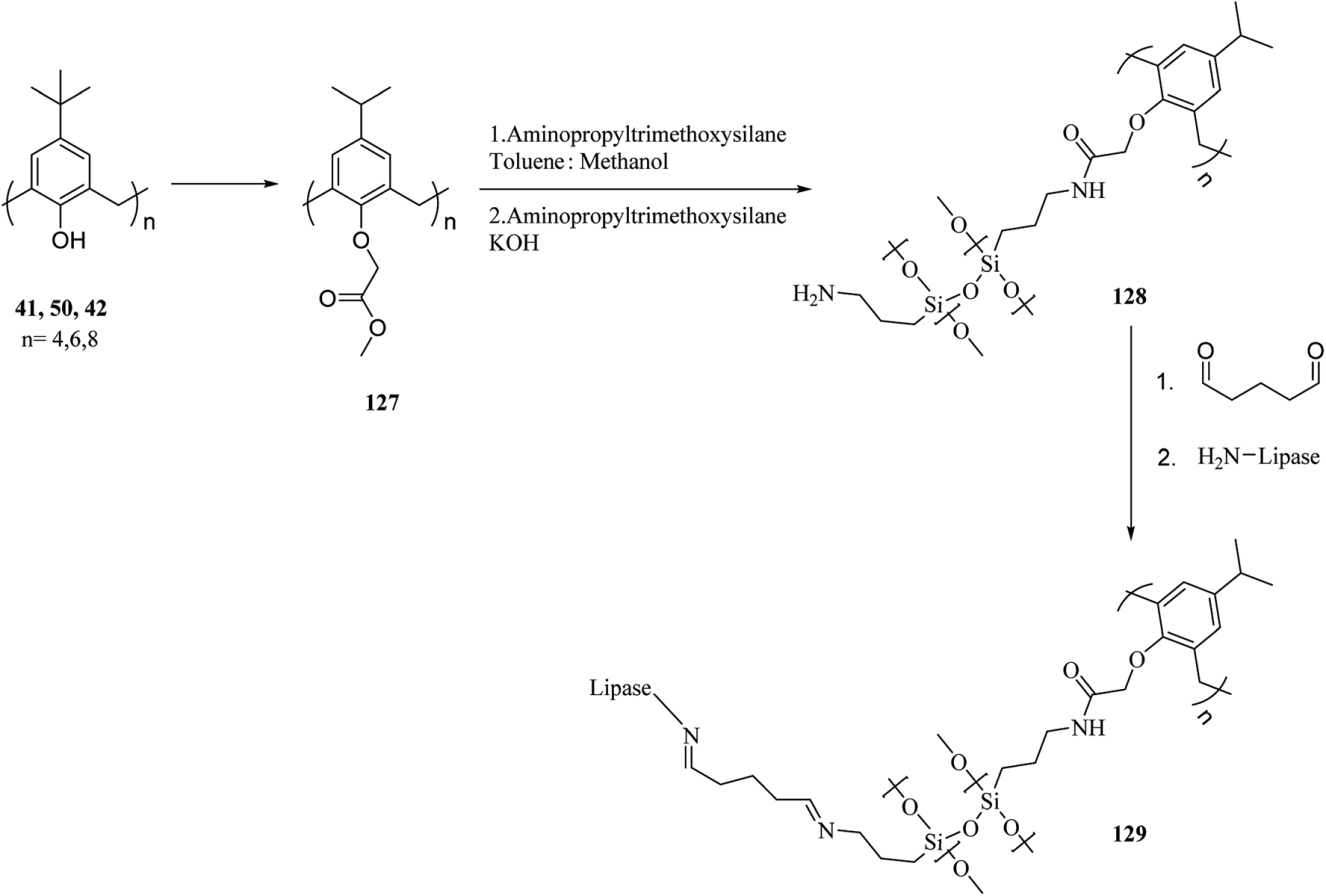
The synthesis of calix[*n*]arene-based silica polymers and immobilization of Candida rugosa lipase.

**Scheme 39:**
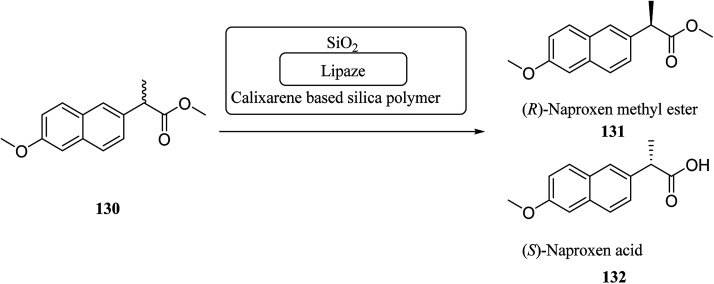
Enantioselective hydrolysis reaction of racemic naproxen methyl ester by encapsulated lipases.

A top priority of enzyme technology is to improve the conformational stability of the enzyme. Erdemir *et al.* examined new calix[*n*]arene-based silica polymers to immobilize candida rugosa lipase (Scheme 38). In addition, covalent attachment of candida rugosa lipase was achieved by the amino-functionalized calix[4, 6, 8]arene-based silica polymers 128 through glutaraldehyde as a coupling agent. The hydrolytic activities of immobilized lipase were examined and compared with the free enzyme. As the results showed, the immobilized lipase has better stability, adaptability, and reusability. Alkoxysilane precursors and calix[*n*]arene based silica polymers encapsulated candida rugosa lipase in sol–gel matrix. The encapsulated lipase was also utilized in the enantioselective hydrolysis reaction of racemic Naproxen methyl ester 130 (Scheme 39).^104,105^

**Scheme 40:**
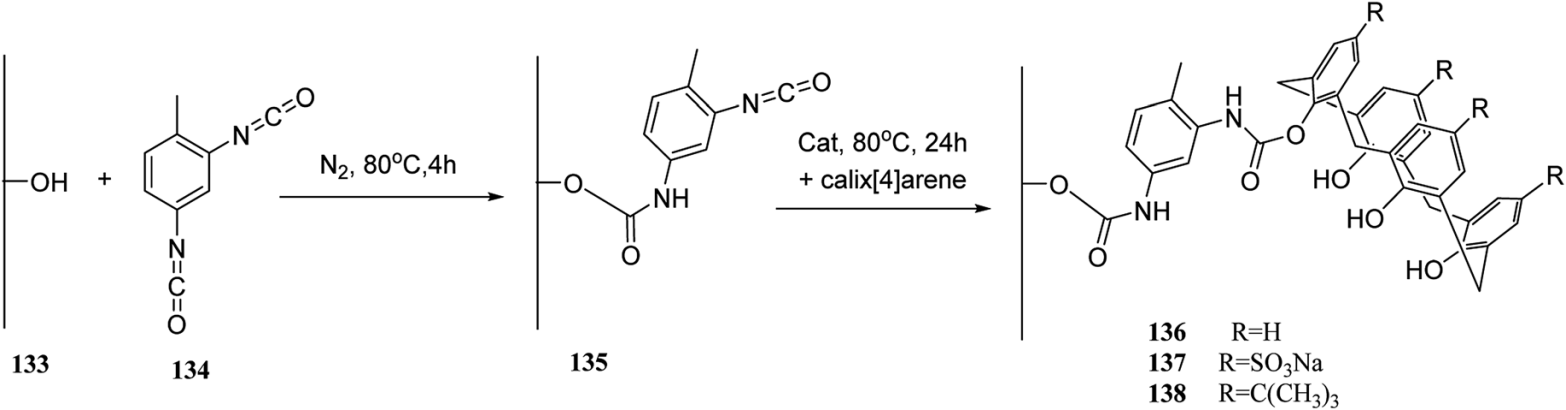
Preparation of mesoporous hybrid materials based on calixarene.

**Scheme 41:**
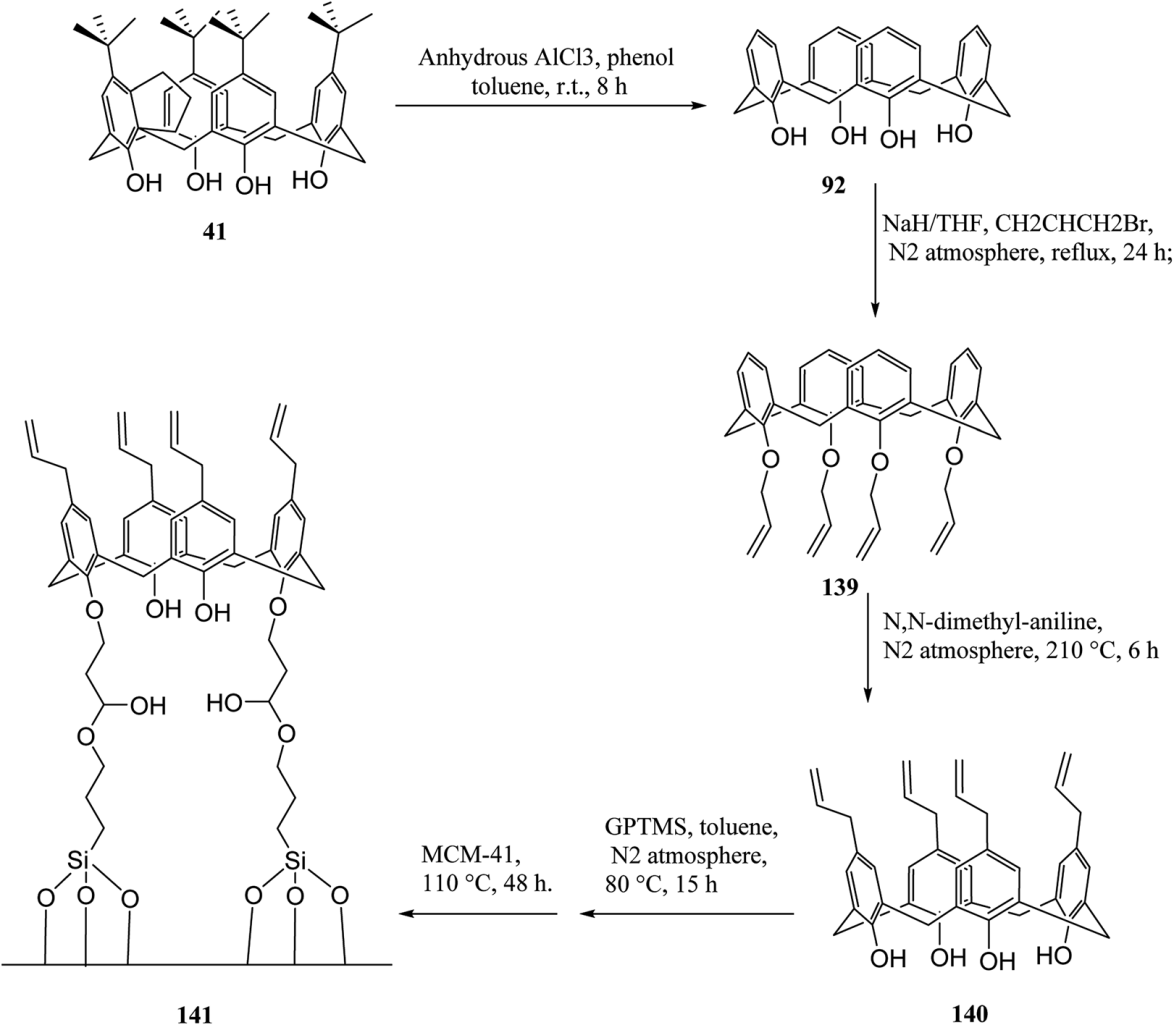
Synthesis of allyl calixarene-functionalized mesoporous silica.

**Scheme 42:**
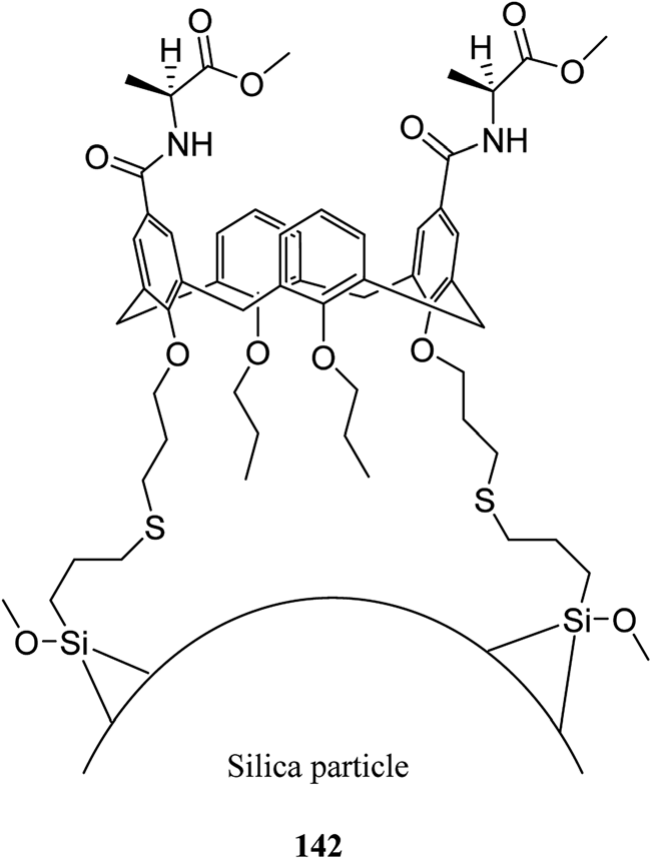
Structure of alanine-calix[4]arene stationary phase.

In another study, Alahmadi *et al.* attached calix[4]arene derivatives covalently onto mesoporous silica 133 using a di-isocyanate 134 as the linker. They utilized toluene 2,4-di-iso-cyanate to create a bridge between mesoporous silica and calix[4]arene derivative (Scheme 40). Afterward, the materials were utilized to examine the sorption properties of organotin compounds tributyltin (TBT), triphenyltin (TPT) and dibutyltin (DBT).^106,107^ MCM allyl calix 141, as another inorganic–organic hybrid material, was synthesized by Narkhede *et al.* using covalent modification of an MCM-41 with a tetra-allyl calixarene 140 conjugate. Scheme 41 demonstrates the synthesis of allyl calixarene functionalized mesoporous silica. The use of MCM-allyl calix hybrid for loading and *in vitro* release of doxorubicin in phosphate-buffered saline and in the cancer cells has been illustrated by studies. As shown by the results, the inorganic–organic hybrid can be used for sustained drug delivery to cancer cells.^108^ The *p-tert*-butyl calix[4]arene mesoporous silica molecular sieves were prepared by Huang *et al.* They also studied diethylstilbestrol and bisphenol A adsorption.^109^ Li *et al.* tried to prepare and assess a new calix[4]arene-bonded silica gel stationary phase for HPLC. They synthesized the stationary phase and conducted the fast analysis for hydrocarbons, which showed considerable selectivity for the sulfonamides.^110^ Paiva *et al.* studied calixarene-functionalized silica gel as microwave-assisted multicomponent synthesis of julolidines. To this end, they used silica-supported calix[4]arene as heterogeneous catalyst.^111^ Feng *et al.* also examined wettability recognition for isomeric phenylene diamine using nitro-calix[4]arene, which was linked to silica gel through click chemistry.^112^ Yabushita *et al.* studied selective sequestration of aromatics out of aqueous mixtures along with sugar using hydrophobic molecular calixarene cavities grafted on silica.^113^ A novel calix[4]arene-based chiral stationary phase was synthesized and the enantio-separation performance was examined through separating the enantiomers of 3,5-dinitrobenzoyl amino acids, diclofop-methyl and d,l-mandelic acid. Scheme 42 illustrates the structure of alanine-calix[4]arene stationary phase 142 which the allyl groups at the lower rim of calix[4]arene were covalently bonded to silica gel using thiol–ene click chemistry reaction.^114^ Finally, Hu *et al.* tried to investigate the retention mechanisms of benzenediol and naphthol positional isomer on a calix[4]arene column using quantum chemistry calculations.^115^

### 3.5 Calixarene-functionalized chitosan

**Scheme 43:**
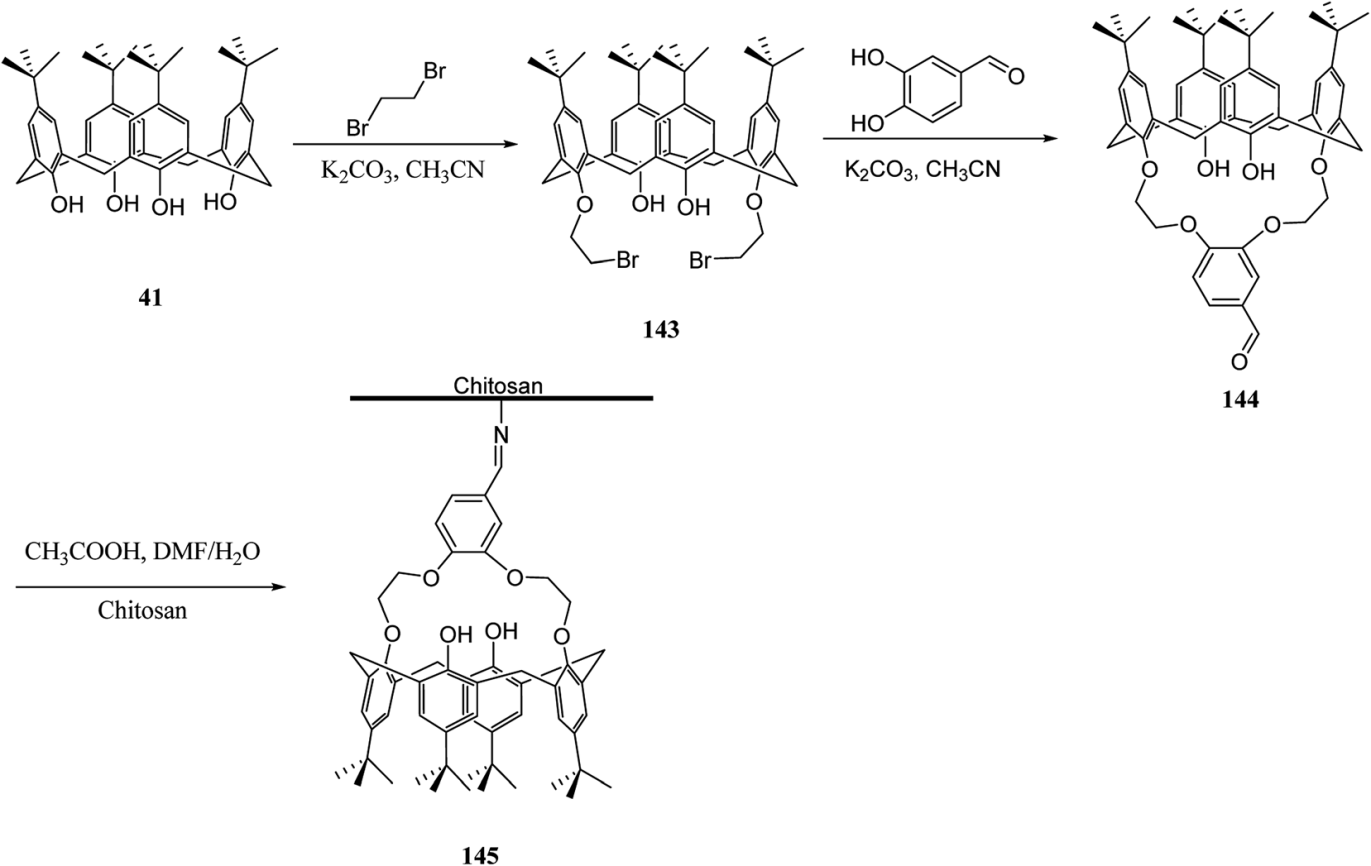
General synthesis of calix[4]crown-grafted chitosan chelating polymer 145.

**Scheme 44:**
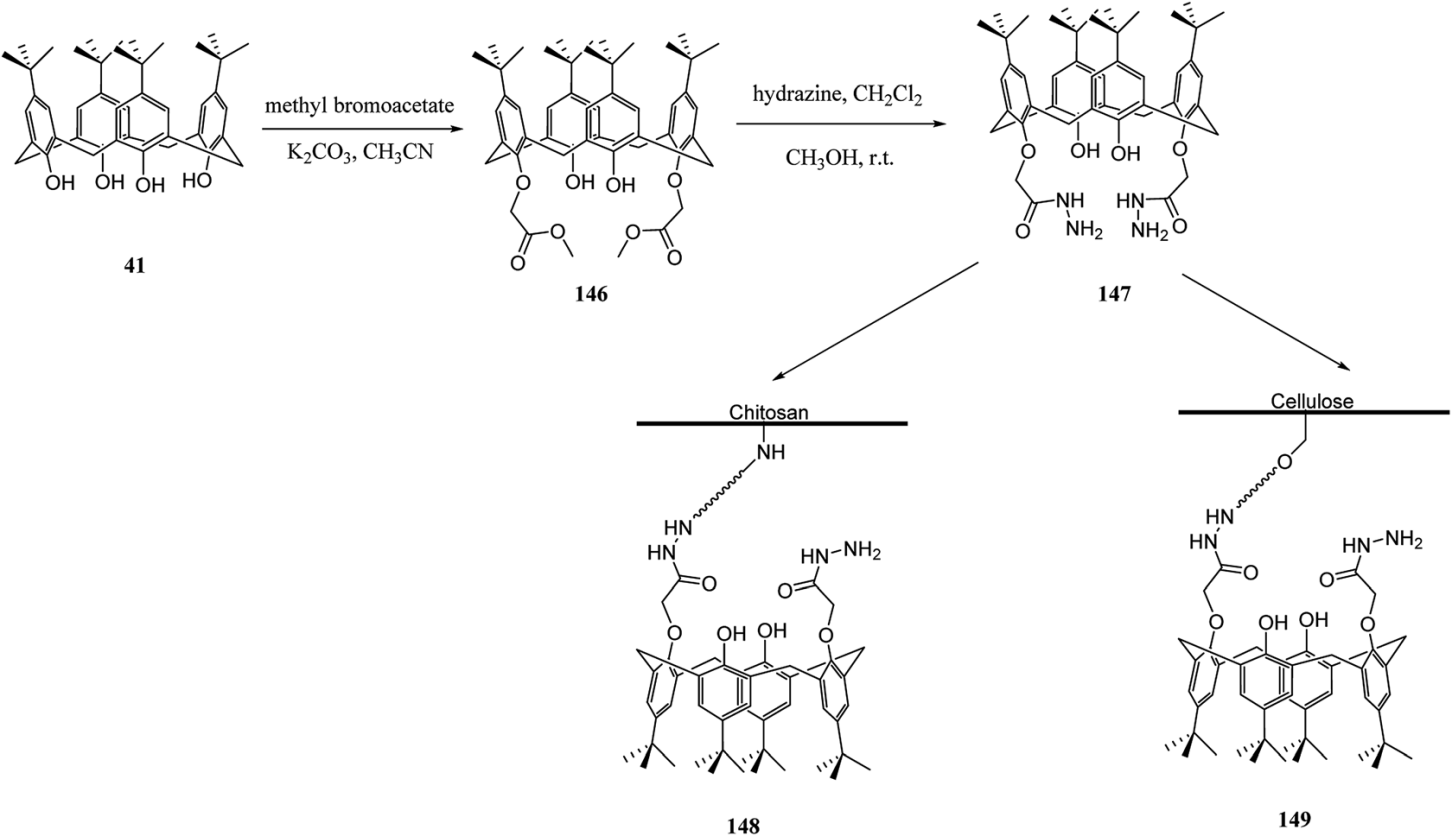
Immobilization of calixarene derivative 147 onto cellulose and chitosan biopolymers.

Several studies have selected chitosan as a polymer to be functionalized with calixarenes.^116,117^ There is a strong adsorption and chelating potential to metal ions in chitosan; however, its selectivity adsorption is not promising. By modifying calix[*n*]arenes with chitosan, we can increase the adsorption capacity of the novel polymers towards metal ions. The reason for this is the synergic action between chitosan and calix[*n*]arenes.^118,119^ For example, Liu *et al.* studied the synthesis and characterizing new calixarene–chitosan polymer and then the extraction efficiency of the obtained polymer was examined for heavy metals, dichromate anions, and rare metals.^120,121^ Calixarene–chitosan polymers were also studied by Tabakci *et al.* and the adsorption property of them was measured. The results revealed that the adsorption capacity of calix[4]arene–chitosan polymers towards specific heavy metal cations (Co^2+^, Hg^2+^, Cu^2+^, Cd^2+^, Pb^2+^, Ni^2+^ and Cr_2_O_7_^2−^) outperformed chitosan. In particular, the adsorption property towards Cr_2_O_7_^2−^ at pH 1.5 was impressive.^122^ Yanagi *et al.* studied chitosan beads featured with upper/lower-rim substituted water-soluble calixarenes as an adsorbent for di-*n*-butyl phthalate (DBP). Using *p*-sulfonatocalix[6]arene and *p*-sulfonatocalix[8]arene could include DBP in their hydrophobic cavities in the aqueous phase. The amount of DBP adsorbed by chitosan beads modified with *p*-sulfonatocalix[6]arenes was approximately five times as large as that for unmodified chitosan beads^123^ In order to remove the dyes from wastewaters, several methods such as precipitation, membrane processes, biological treatment and adsorption, *etc.* are applied to the treatment of dye-containing effluent. Among them, adsorption is one of the most efficient methods due to its simple, fast, selective separation and reusable process. As a result, the design and synthesis of novel dye absorbent is an important issue in this area. To this end, Fang *et al.* examined the synthesis and dyes adsorption properties of calix[4]crown-grafted chitosan 145 chelating polymer. Scheme 43 illustrates the synthetic route of the target polymer.^124^ Moreover, Lu *et al.* synthesized a new calix[4]arene derivatized chitosan bonded stationary phase for high-performance liquid chromatography (HPLC). Its chromatographic performance and retention mechanism were evaluated using different solute including mono-substituted benzenes, phenols and nucleosides. The results showed that the desired stationary phase could provide various interactions with solutes, such as hydrophobic, hydrophilic, π–π, and inclusion interactions.^125^ Ozyilmaz *et al.* also studied the process of preparing new calixarene biopolymers including immobilization of calixarene derivative 147 onto cellulose and chitosan biopolymers. They also examined the encapsulation of these calixarene biopolymers using candida rugosa lipase (Scheme 44).^126^

### 3.6 Other studies related to polymers functionalized *via* calixarene moieties

**Scheme 45:**
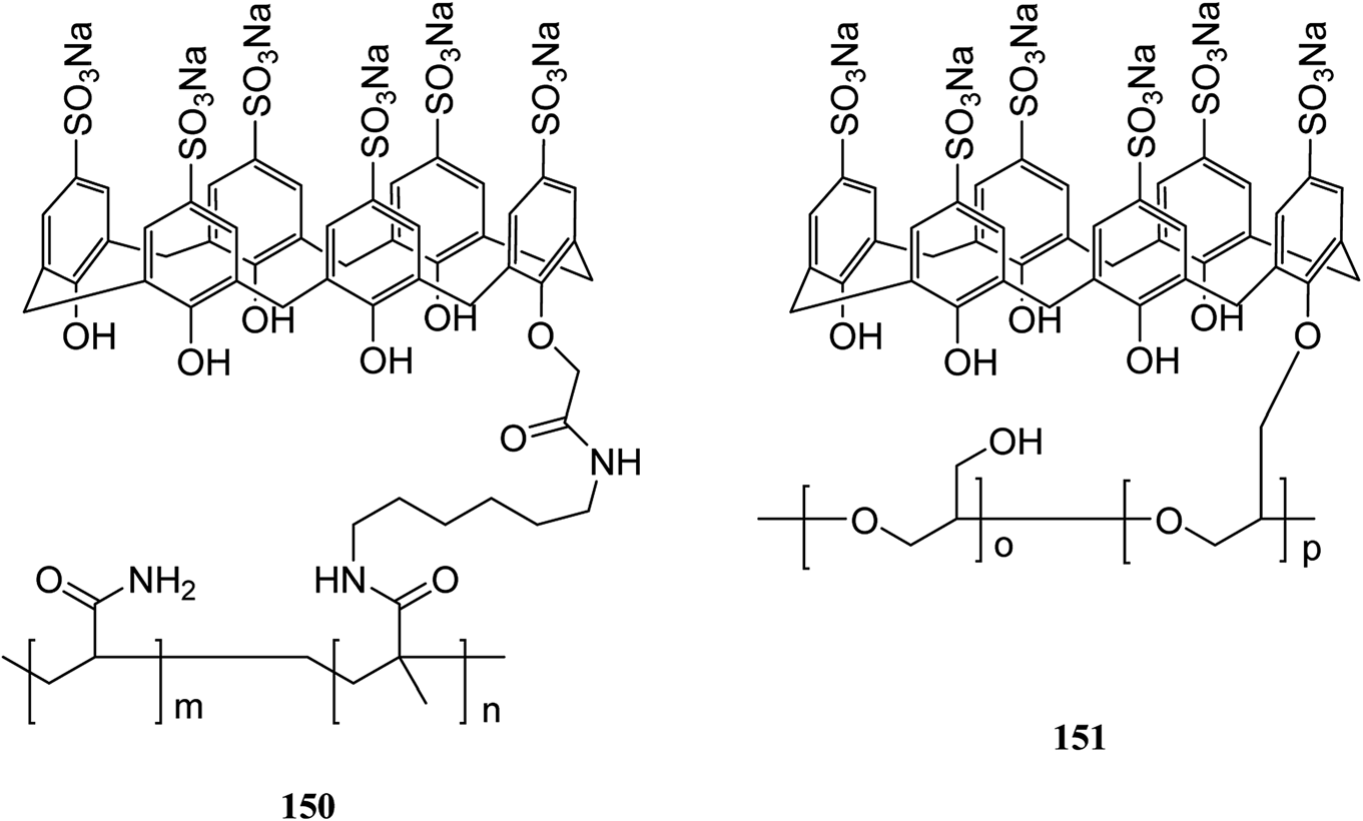
The representation of calixarene-functionalized polymers 150 and 151.

Immobilization of calixarenes on a polymer support and resins, such as Wang, Merrifield, silica, chitosan and cellulose, has been reported in the literatures as a means for the desirable goals. So far, we reviewed several important examples of them. In this section, we discuss other studies related to the polymers functionalized *via* calixarene moieties. For instance, Chen *et al.* studied adsorption and desorption specifications of the starch grafted *p-tert*-butyl-calix[*n*]arenes (SGC4, SGC6, SGC8) as a calixarene-functionalized biopolymer for butyl rhodamine B (BRB) solution as a simulated dye wastewater. The adsorption of BRB onto SGC8 is better represented by the Langmuir equation and as the result, new-style adsorbent of SGC8 is regarded as a potential adsorbent to deal with dye or organic wastewater.^127^ Cross-linking of (sodium calix[6]arenehexasulfonic acid) SCX6 with epichlorohydrin or the covalent binding of carboxymethylated SCX6 to a vinyl polymer carrying a pendant primary amino group were used by Kitano *et al.* to introduce two novel calixarene-functionalized polymers 150 and 151 (Scheme 45). Fluorimetry studies have examined the complexation of bisphenol A with the SCX6 group in the polymers.^128^ A new material through a reaction between a calix[4]arene amine derivative and an oxidized activated carbon was also synthesized by Toumi *et al.* The results indicated that there was a link between oxygen functional group and calixarene *via* covalent bond. This first attempt of grafting calixarene on activated carbon paves the way for grafting different kinds of calixarenes on this support. The efficiency and selectivity of the extraction properties of this new material towards metal ions are presently under investigation.^129^

**Scheme 46:**
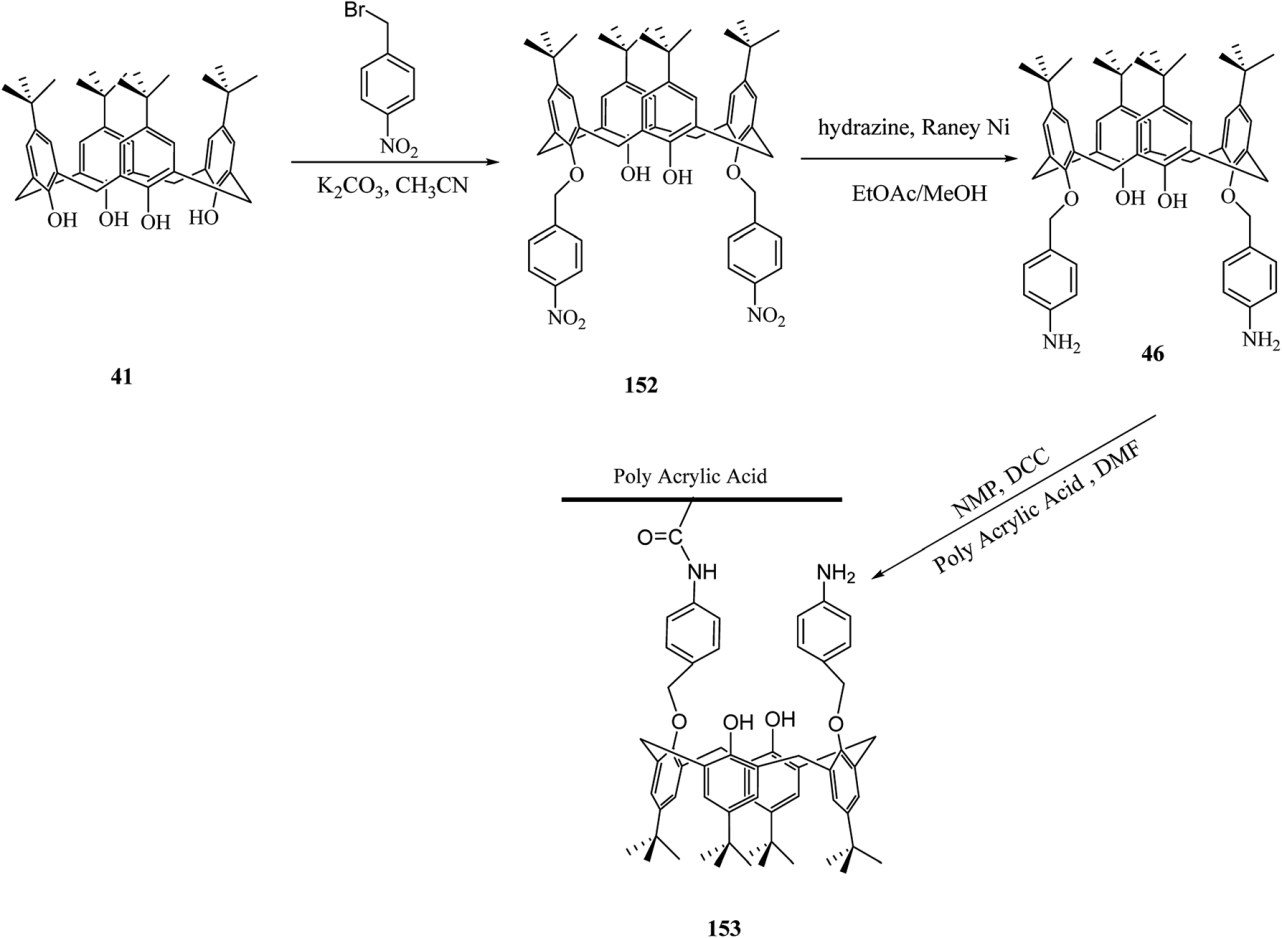
Modification of poly acrylic acid (PAA) using calix[4]arene diamine 46.

The development of novel sensors such as calixarene-based materials for the detection of heavy metals were investigated in many studies. Toxic heavy metals are dangerous because of their accumulative and persistent character in the environment. Because of this reason, Hajipour *et al.* studied the modification of poly acrylic acid (PAA) using calix[4]arene derivatives for the adsorption of toxic heavy metals. For example calix[4]arene diamine derivative 46 was synthesized and then grafted to PAA (Scheme 46).^130^

**Scheme 47:**
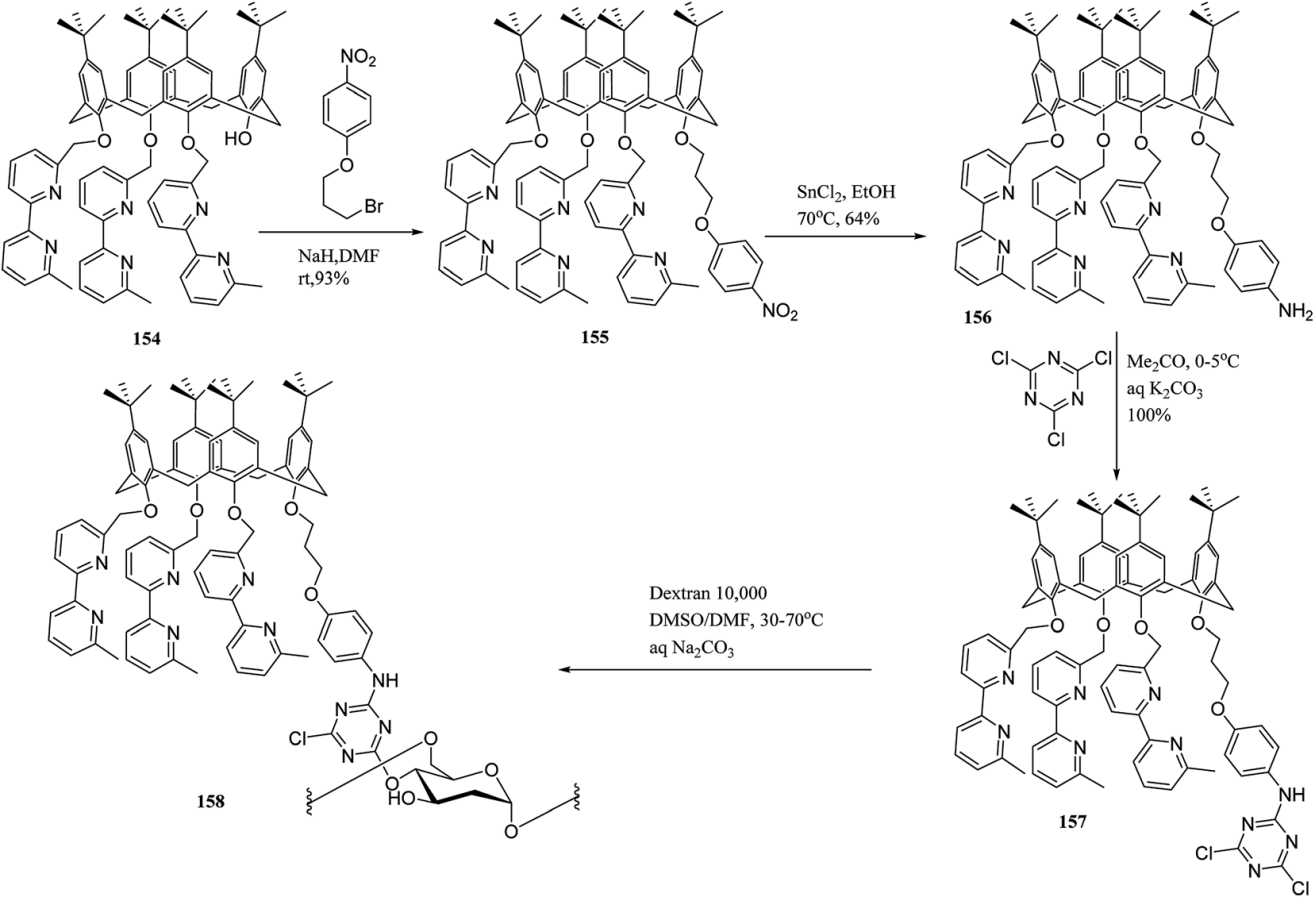
Immobilization of bifunctional calix[4]arene 157 on carbohydrate polymers.

Also, Trivedi *et al.* synthesized calix[6]hydroxy amide-based polymer, which is useful to trap the metal ions of uranium, thorium, and cerium.^66^ To graft on natural polymeric materials like water-soluble dextrans and for metal complexation, multi-step synthesis of a novel bifunctional calix[4]arene was designed by Engrand *et al.* (Scheme 47). At the final step, the pendant was anchored to the polymer using a cyanuric linker. The obtained material demonstrated an intriguing capability to complex with copper(i).^131^

**Scheme 48:**
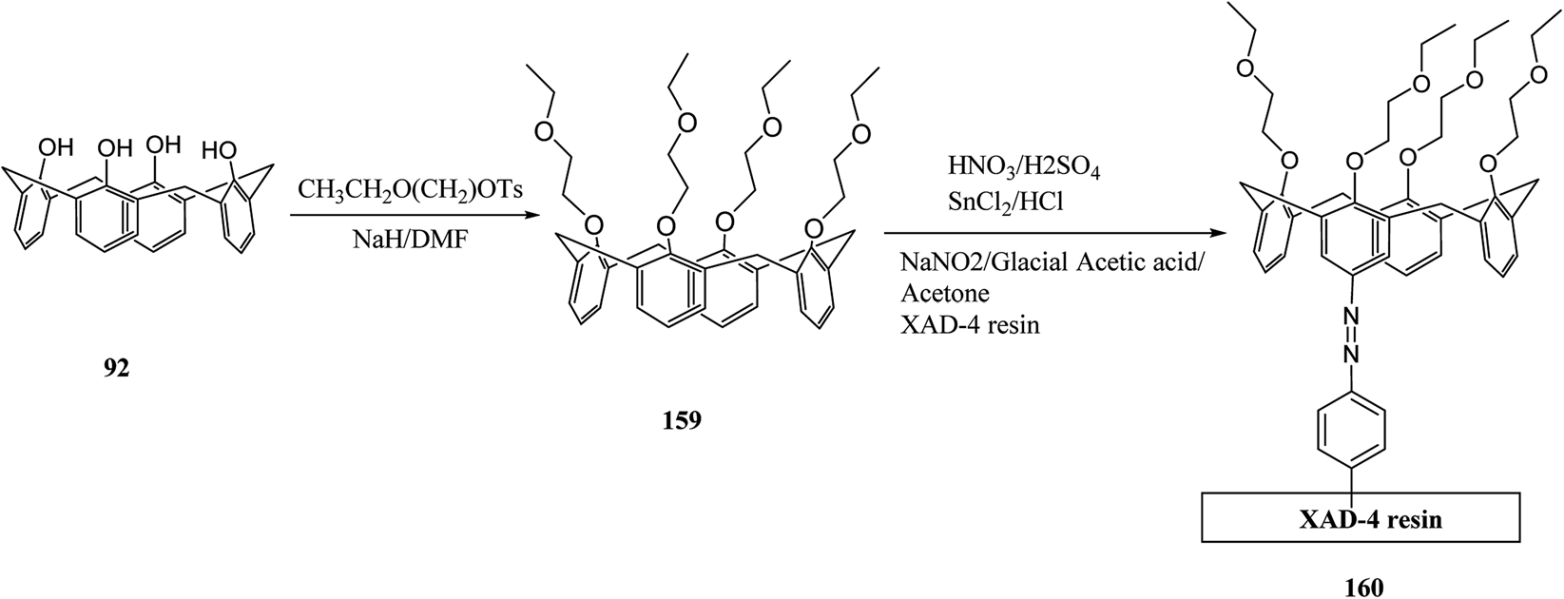
The presentation of calixarene-based functional material 160.

In general, an indicator of the quality of life is the quality of water, which is also a determining factor for the safe public health. An effective calixarene-functionalized material was introduced by Khan *et al.* (through reaction of calix[4]arene derivative 159 with XAD-4 resin) to separate and recover Cr(vi) from water (Scheme 48). Different parameters, like change in pH, concentration, flow rate and bed height were optimized and the highest sorption capacity was equal to 85 mg g^−1^. In addition, with optimum conditions, this calixarene-based functional material was recovered up to 97%.^132^

## 4. Conclusion

In conclusion, as the examples are shown during the recent decade, calixarene-based polymers have drawn growing attention as a platform to build advanced materials. Here we reviewed many interesting studies related to polymers based on calixarenes. The selected examples presented in this review demonstrated that calixarene-based polymers have a higher concentration of the binding sites rather than single calixarene derivatives, which can significantly improve their supramolecular properties. Furthermore, the immobilization of calixarene derivatives on solid supports opens up a wide horizon of opportunities for important applications such as water purification and removal of environmental pollutions. On the other hand, synthesis of calixarene-based polymers is usually complicated and methods of purification are often difficult. Low yield and poor solubility are other limiting factors of these polymers which still exist. Therefore, it is clear that further progress in this field requires more improvements in methodologies and development of new calixarene-containing monomers and also versatile calixarene derivatives.

## Conflicts of interest

There are no conflicts to declare.

## Supplementary Material
